# Supplementary material for: Serological Response Patterns to Assess Treatment Outcomes in Advanced Non-Small Cell Lung Cancer: A Real-World Exploratory Multi-Center Observational Cohort Study
Source: Cancers (Basel). 2025 Nov 13;17(22):3647. doi: 10.3390/cancers17223647 (PMC12651941; doi:10.3390/cancers17223647)
Supplement: Supplementary file 1 [file cancers-17-03647-s001.zip › cancers-3894620-supplementary.pdf]

Article

Serological Response Patterns to Assess Treatment Outcomes in Advanced Non-Small Cell Lung Cancer: A Real-World Exploratory Multi-Center Observational Cohort Study

Materials and Methods

Study design and patient population

We performed a real-world exploratory multi-center observational cohort study that included adults with advanced non-small cell lung cancer (NSCLC) who received immune checkpoint inhibitor (ICI)-containing treatment at the Radboud University Medical Center (Radboudumc), Nijmegen, The Netherlands, or the Netherlands Cancer Institute (NKI), Amsterdam, The Netherlands, between March 2013 and January 2023. ICI-containing treatment was administered in accordance with corresponding study protocols or local guidelines that were applicable at treatment start (e.g. early access, compassionate use program, clinical trials, routine care). Patients were included if they had disease control three months after treatment start, and if at least three serum tumor marker (STM) measurements for at least one STM had been performed during treatment. Clinical disease control was defined as no deterioration or an improvement in clinical symptoms as assessed by the treating thoracic oncologist [1]. Radiological disease control was defined as stable disease (SD), partial response (PR), or complete remission (CR) based on tumor evaluation by the radiologist on Computed Tomography (CT)-imaging according to Response Evaluation Criteria in Solid Tumors (RECIST) version 1.1 [2]. Importantly, STM measurements had to be performed between baseline and seven days after the maximum treatment period of two years (if clinical and/or radiological progression had not occurred between three months and two years after treatment start (defined as having achieved a durable response)) or the date of progression (if clinical and/or radiological progression had occurred between three months and two years after treatment start (defined as having developed secondary treatment resistance)) [2,3]. Baseline was defined as between three weeks before and seven days after treatment start to include as many patients as possible. Furthermore, at least one of the STM measurements had to be performed between 84 days (corresponding to two treatment cycles of six weeks, four treatment cycles of three weeks, or six treatment cycles of two weeks) before and seven days after the maximum treatment period of two years (in patients with a durable response) or the date of progression (in patients who developed secondary treatment resistance) to ensure that at least one STM measurement had been performed as close to the date of the maximum treatment period or the progression date as possible, while simultaneously preventing the inclusion of STM measurements performed after the maximum treatment duration if a durable response was achieved or during a subsequent treatment line if secondary treatment resistance had occurred. For patients with a durable response in whom ICI-containing treatment was discontinued early (e.g. due to immune-related adverse events (irAEs)), the date that would have corresponded to the maximum treatment period of two years was used.

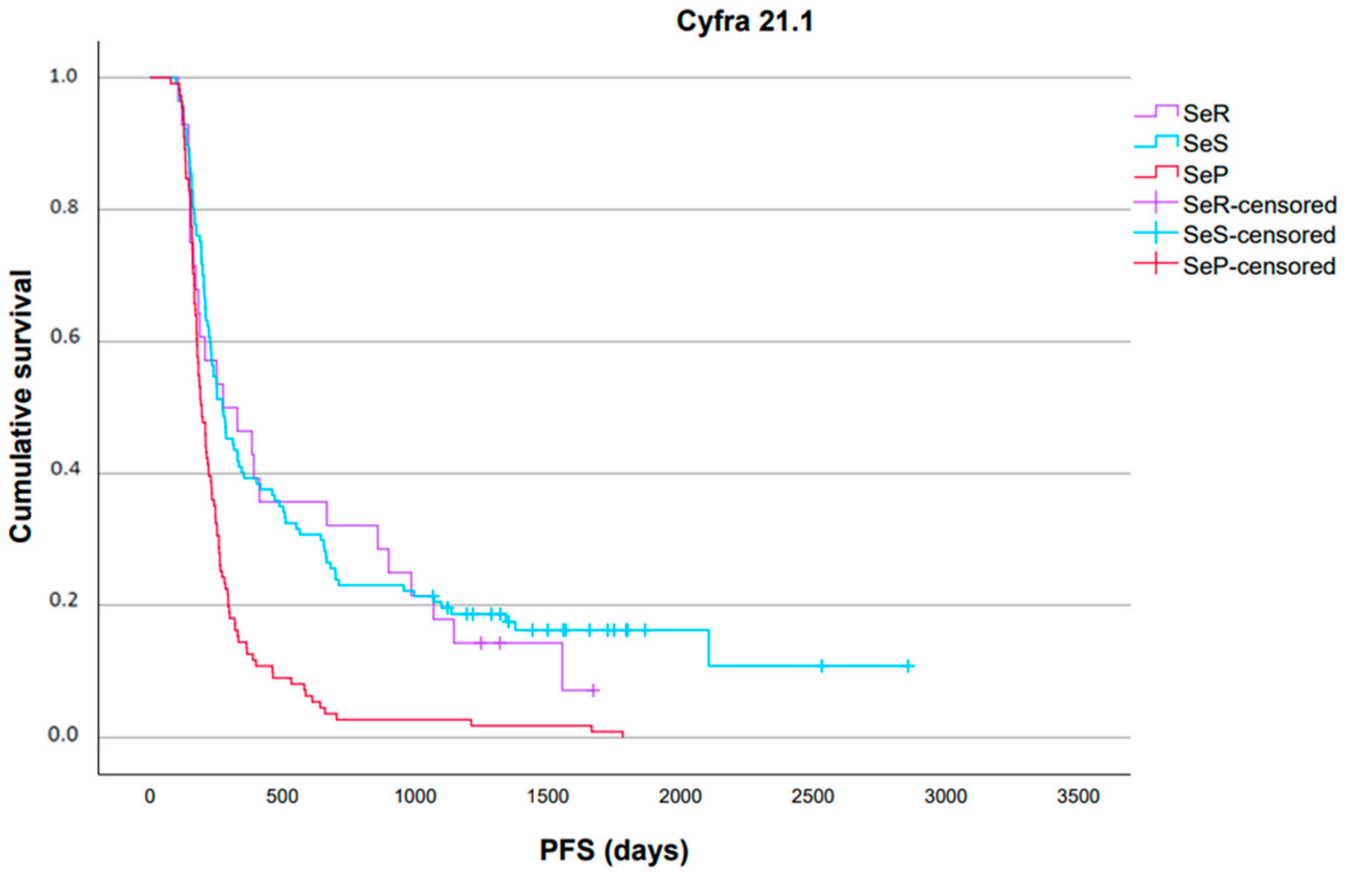

No. at risk								
	**0**	**500**	**1000**	**1500**	**2000**	**2500**	**3000**	**3500**
**SeR**	28 (0)	10 (0)	6 (0)	2 (2)	0 (3)	0 (3)	0 (3)	0 (3)
**SeS**	117 (0)	41 (0)	25 (0)	11 (9)	3 (17)	2 (17)	0 (19)	0 (19)
**SeP**	111 (0)	10 (0)	3 (0)	2 (0)	0 (0)	0 (0)	0 (0)	0 (0)

Figure S1. Progression-free survival associated with the three serological response patterns for Cyfra 21.1. Abbreviations: Cyfra 21.1, Cytokeratin 19 fragment antigen; SeR, serological remission; SeS, serological stable/unknown significance; SeP, serological progression; PFS, progression-free survival; No., number.

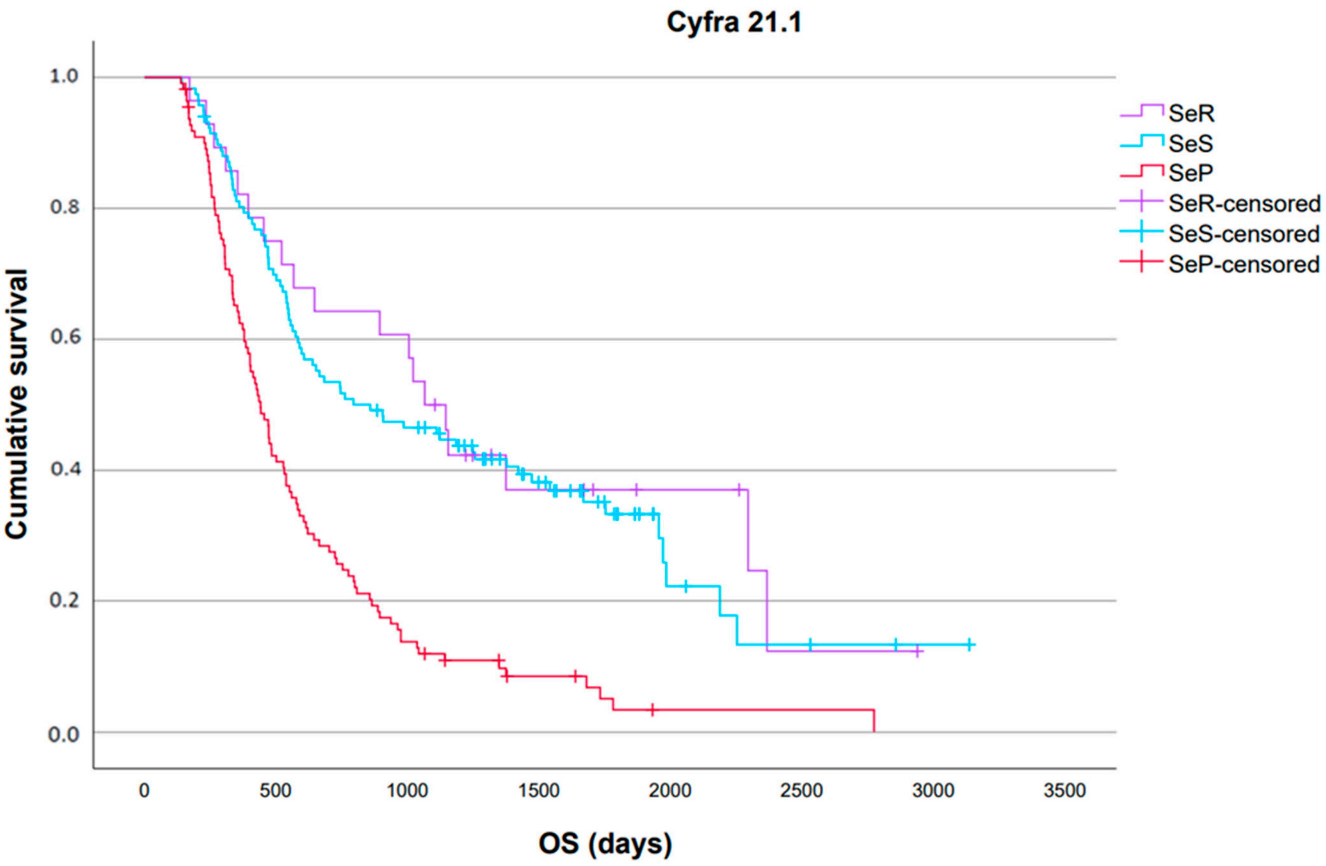

No. at risk								
	**0**	**500**	**1000**	**1500**	**2000**	**2500**	**3000**	**3500**
**SeR**	28 (0)	21 (0)	17 (0)	7 (4)	4 (7)	1 (8)	0 (9)	0 (9)
**SeS**	117 (0)	81 (1)	53 (2)	30 (17)	6 (35)	3 (36)	1 (38)	0 (39)
**SeP**	111 (0)	46 (2)	15 (2)	6 (6)	1 (8)	1 (8)	0 (8)	0 (8)

Figure S2. Overall survival associated with the three serological response patterns for Cyfra 21.1. Abbreviations: 21. 1, Cytokeratin 19 fragment antigen; SeR, serological remission; SeS, serological stable/unknown significance; SeP, serological progression; OS, overall survival; No., number. .

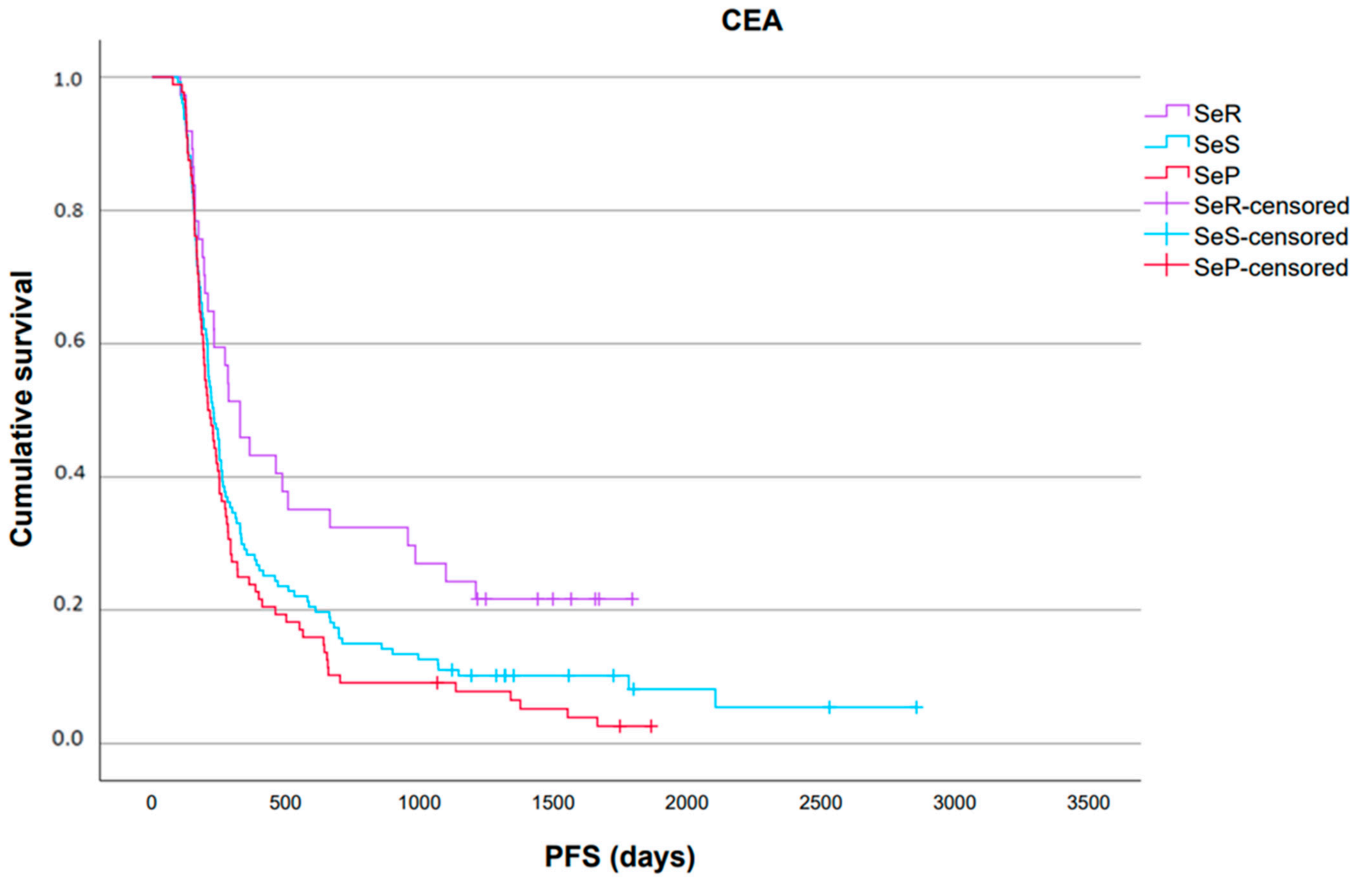

	No. at risk							
	**0**	**500**	**1000**	**1500**	**2000**	**2500**	**3000**	**3500**
**SeR**	37 (0)	14 (0)	10 (0)	4 (4)	0 (8)	0 (8)	0 (8)	0 (8)
**SeS**	127 (0)	30 (0)	16 (0)	7 (6)	3 (9)	2 (9)	0 (10)	0 (11)
**SeP**	88 (0)	17 (0)	8 (0)	4 (1)	0 (3)	0 (3)	0 (3)	0 (3)

Figure S3. Progression-free survival associated with the three serological response patterns for CEA. Abbreviations: CEA, CarcinoEmbryonic Antigen; SeR, serological remission; SeS, serological stable/unknown significance; SeP, serological progression; PFS, progression-free survival; No., number.

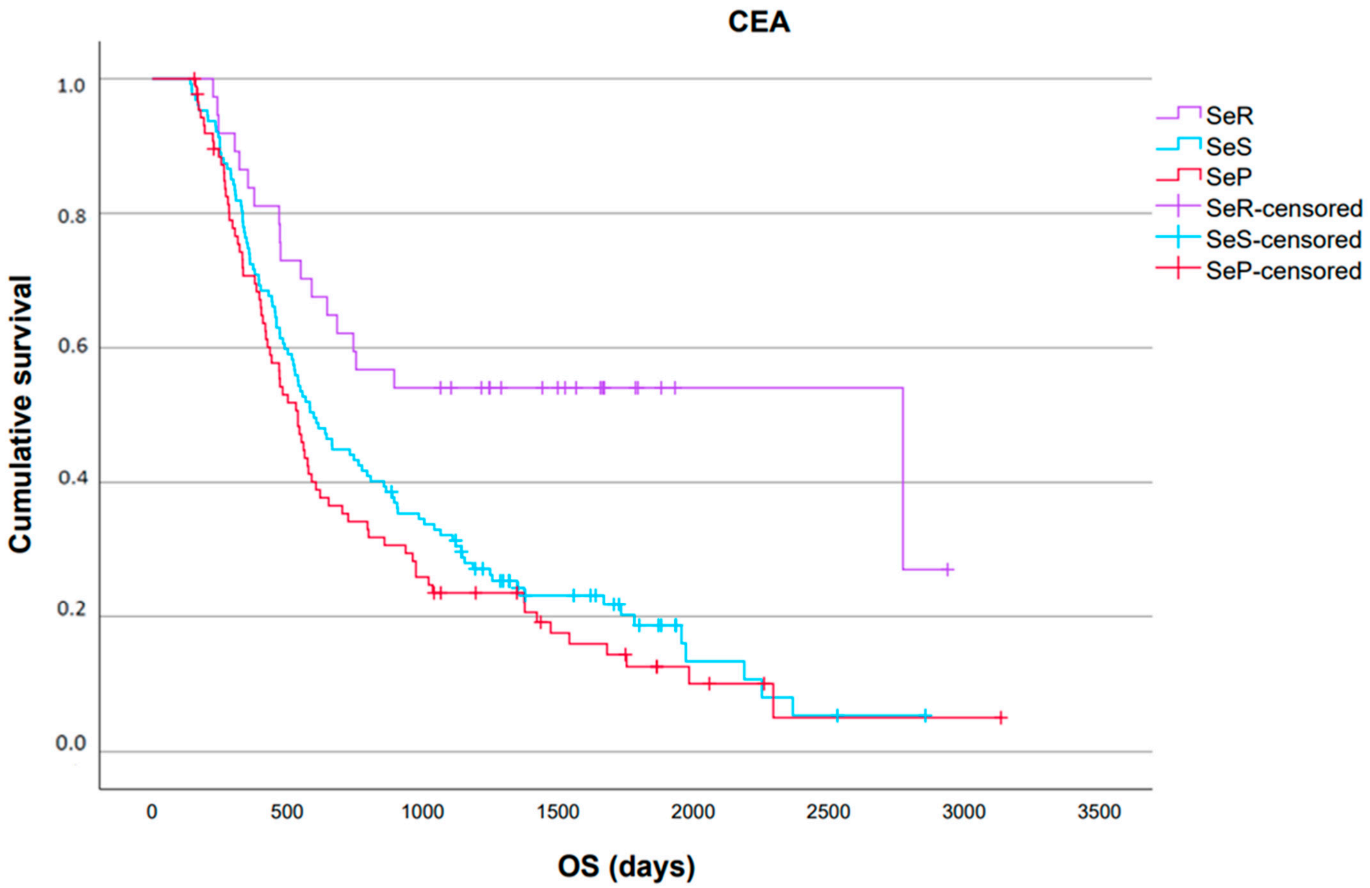

No. at risk								
	**0**	**500**	**1000**	**1500**	**2000**	**2500**	**3000**	**3500**
**SeR**	37 (0)	27 (0)	20 (0)	12 (8)	2 (18)	2 (18)	0 (19)	0 (19)
**SeS**	127 (0)	76 (0)	43 (1)	20 (11)	5 (21)	2 (21)	0 (23)	0 (23)
**SeP**	88 (0)	45 (3)	22 (3)	11 (8)	4 (11)	1 (13)	1 (13)	0 (14)

Figure S4. Overall survival associated with the three serological response patterns for CEA. Abbreviations: CEA, CarcinoEmbryonic Antigen; SeR, serological remission; SeS, serological stable/unknown significance; SeP, serological progression; OS, overall survival; No., number.

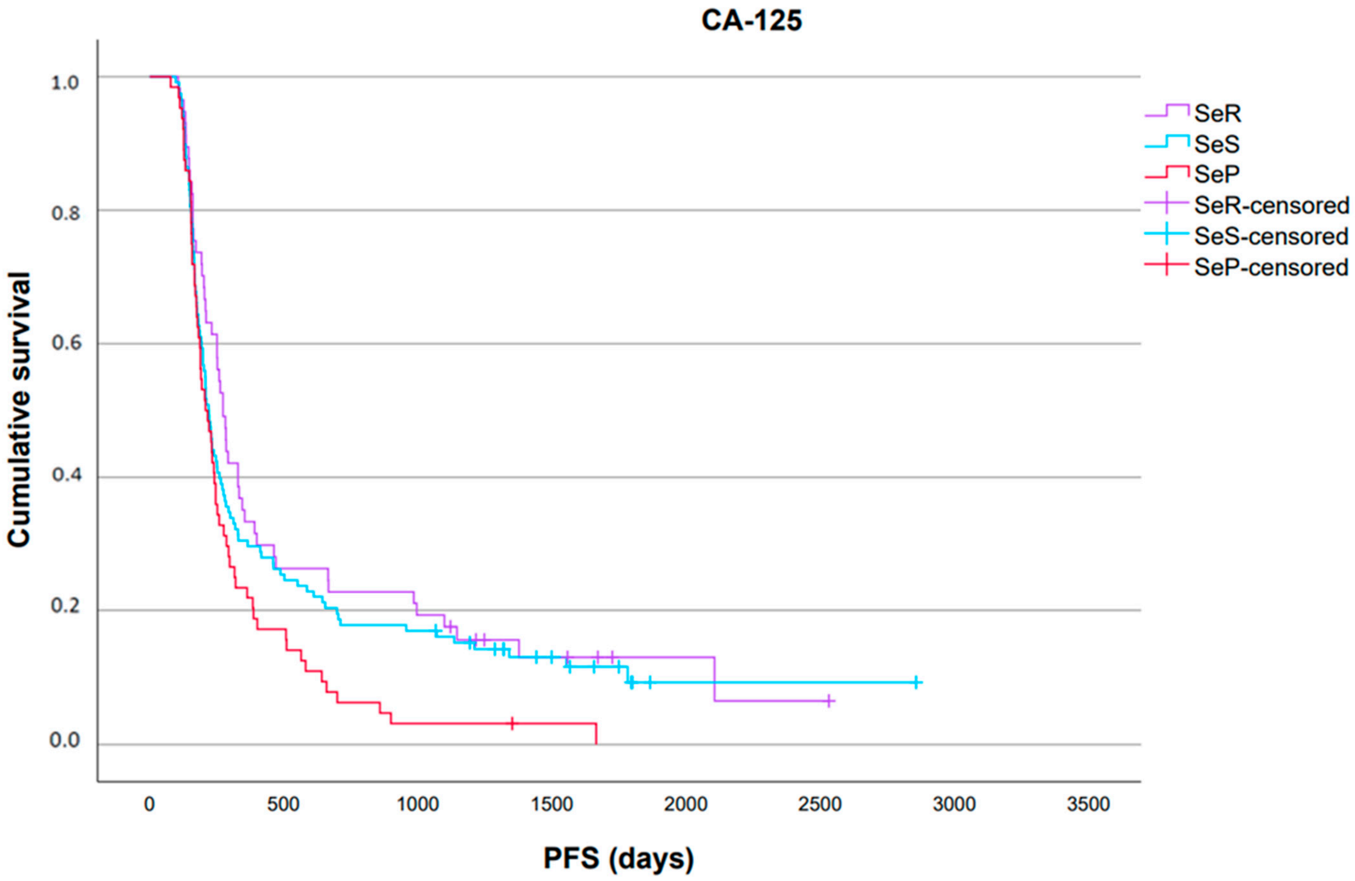

No. at risk								
	**0**	**500**	**1000**	**1500**	**2000**	**2500**	**3000**	**3500**
**SeR**	57 (0)	15 (0)	11 (0)	5 (3)	2 (6)	1 (6)	0 (7)	0 (7)
**SeS**	118 (0)	30 (0)	20 (0)	9 (7)	1 (13)	1 (13)	0 (14)	0 (14)
**SeP**	64 (0)	11 (0)	2 (0)	1 (1)	0 (1)	0 (1)	0 (1)	0 (1)

Figure S5. Progression-free survival associated with the three serological response patterns for CA-125. Abbreviations: CA-125, Cancer Antigen-125; SeR, serological remission; SeS, serological stable/unknown significance; SeP, serological progression; PFS, progression-free survival; No., number.

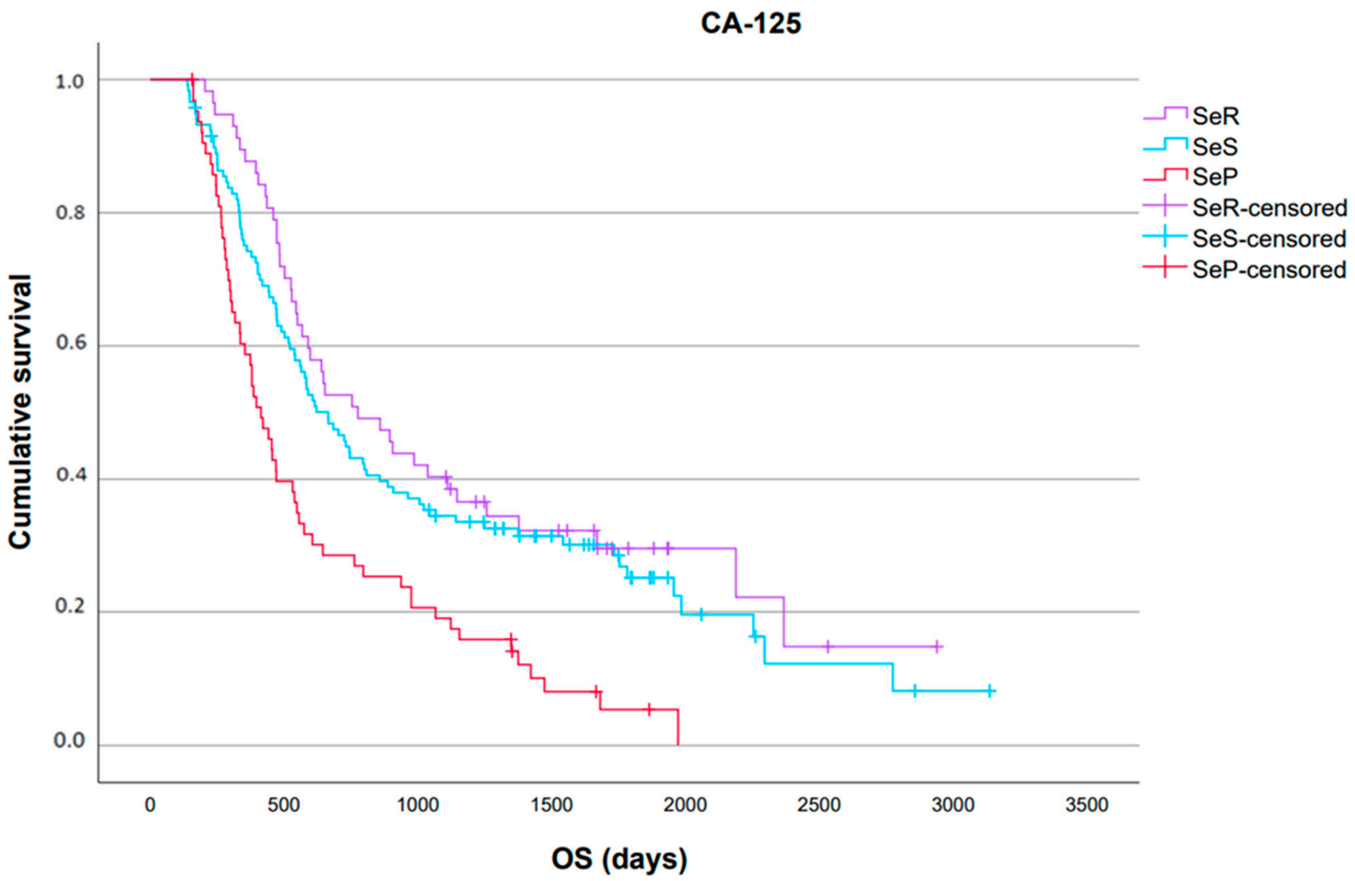

No. at risk								
	**0**	**500**	**1000**	**1500**	**2000**	**2500**	**3000**	**3500**
**SeR**	57 (0)	41 (0)	24 (0)	15 (4)	4 (14)	2 (14)	0 (16)	0 (16)
**SeS**	118 (0)	72 (2)	43 (2)	24 (15)	7 (26)	3 (28)	1 (29)	0 (30)
**SeP**	64 (0)	25 (1)	13 (1)	4 (3)	0 (5)	0 (5)	0 (5)	0 (5)

Figure S6. Overall survival associated with the three serological response patterns for CA-125. Abbreviations: CA-125, Cancer Antigen-125; SeR, serological remission; SeS, serological stable/unknown significance; SeP, serological progression; OS, overall survival; No., number.

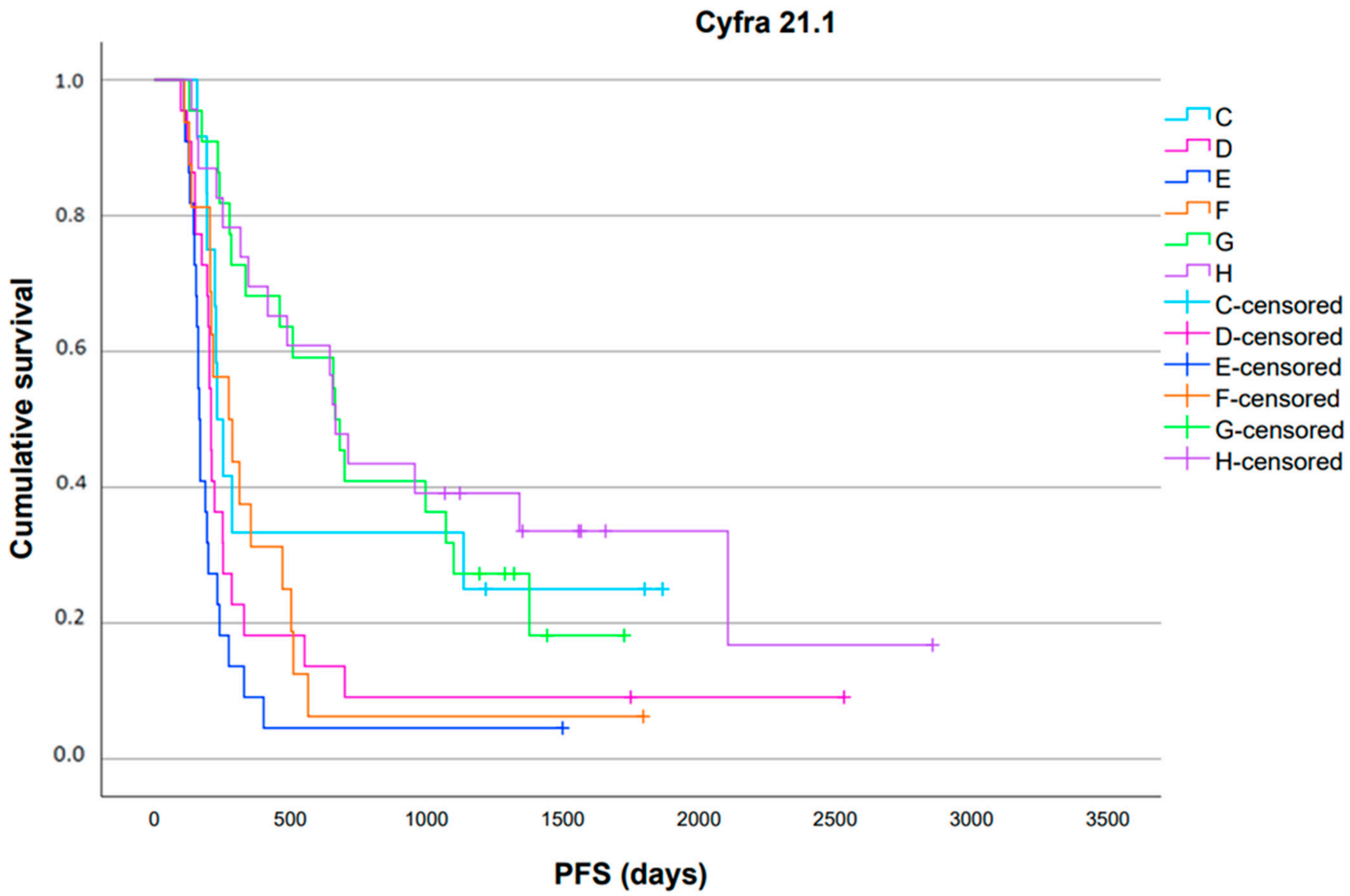

No. at risk								
	**0**	**500**	**1000**	**1500**	**2000**	**2500**	**3000**	**3500**
**C**	12 (0)	4 (0)	4 (0)	2 (1)	0 (3)	0 (3)	0 (3)	0 (3)
**D**	22 (0)	4 (0)	2 (0)	2 (0)	1 (1)	1 (1)	0 (2)	0 (2)
**E**	22 (0)	1 (0)	1 (0)	0 (1)	0 (1)	0 (1)	0 (1)	0 (1)
**F**	16 (0)	4 (0)	1 (0)	1 (0)	0 (1)	0 (1)	0 (1)	0 (1)
**G**	22 (0)	14 (0)	8 (0)	1 (4)	0 (5)	0 (5)	0 (5)	0 (5)
**H**	23 (0)	14 (0)	9 (0)	5 (3)	2 (6)	1 (6)	0 (7)	0 (7)

Figure S7. Progression-free survival associated with the STM dynamics subclassified as SeS for Cyfra 21.1. Abbreviations: STM, serum tumor marker; Cyfra 21.1, Cytokeratin 19 fragment antigen; SeS, serological stable/unknown significance; PFS, progression-free survival; No., number.

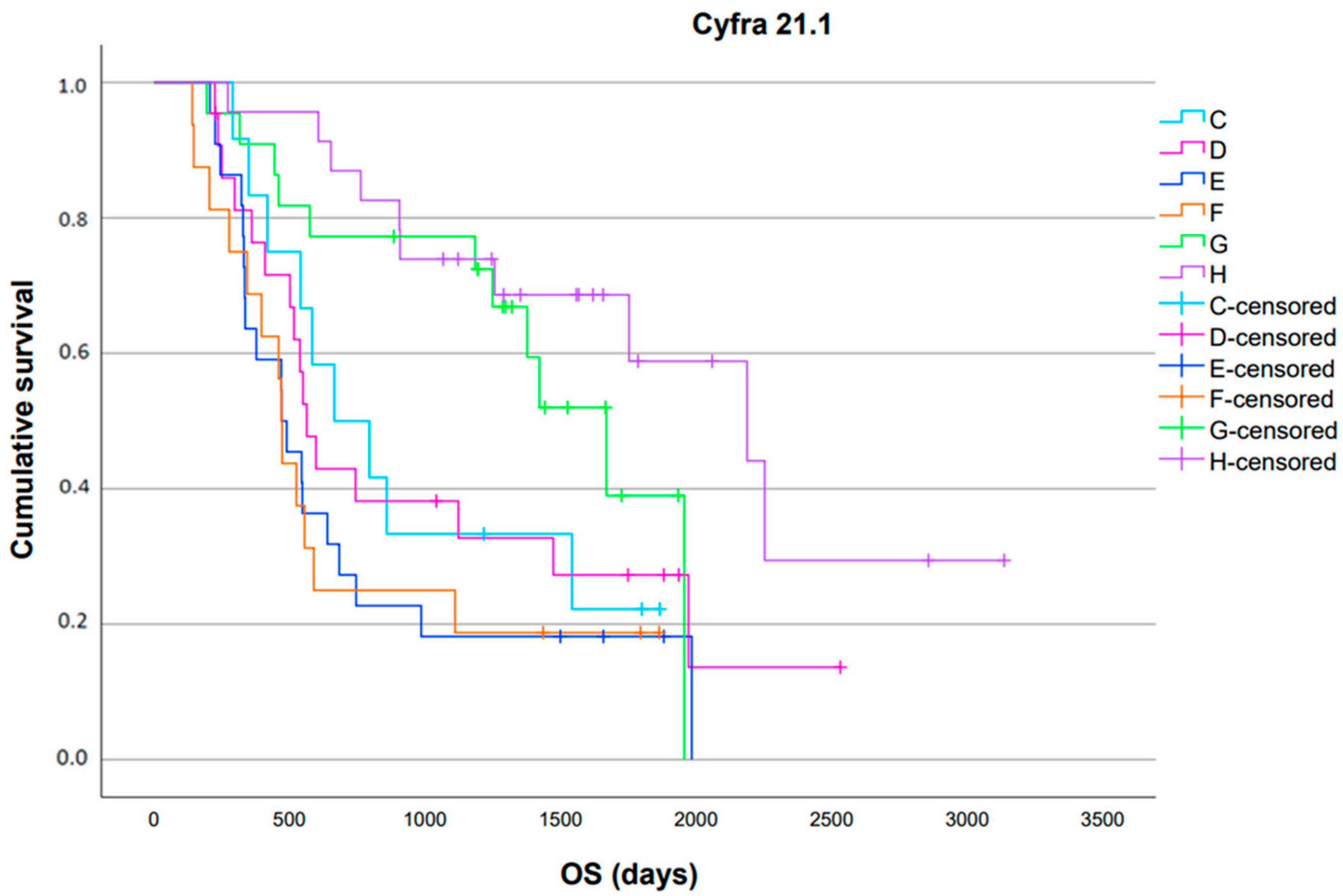

No. at risk								
	**0**	**500**	**1000**	**1500**	**2000**	**2500**	**3000**	**3500**
**C**	12 (0)	9 (0)	4 (0)	3 (1)	0 (3)	0 (3)	0 (3)	0 (3)
**D**	22 (0)	15 (1)	8 (1)	5 (2)	1 (5)	1 (5)	0 (6)	0 (6)
**E**	22 (0)	10 (0)	4 (0)	3 (1)	0 (3)	0 (3)	0 (3)	0 (3)
**F**	16 (0)	7 (0)	4 (0)	2 (1)	0 (3)	0 (3)	0 (3)	0 (3)
**G**	22 (0)	18 (0)	16 (1)	6 (7)	0 (11)	0 (11)	0 (11)	0 (11)
**H**	23 (0)	22 (0)	17 (0)	11 (5)	5 (10)	2 (11)	1 (12)	0 (13)

Figure S8. Overall survival associated with the STM dynamics subclassified as SeS for Cyfra 21.1. Abbreviations: STM, serum tumor marker; Cyfra 21.1, Cytokeratin 19 fragment antigen; SeS, serological stable/unknown significance; OS, overall survival; No., number. .

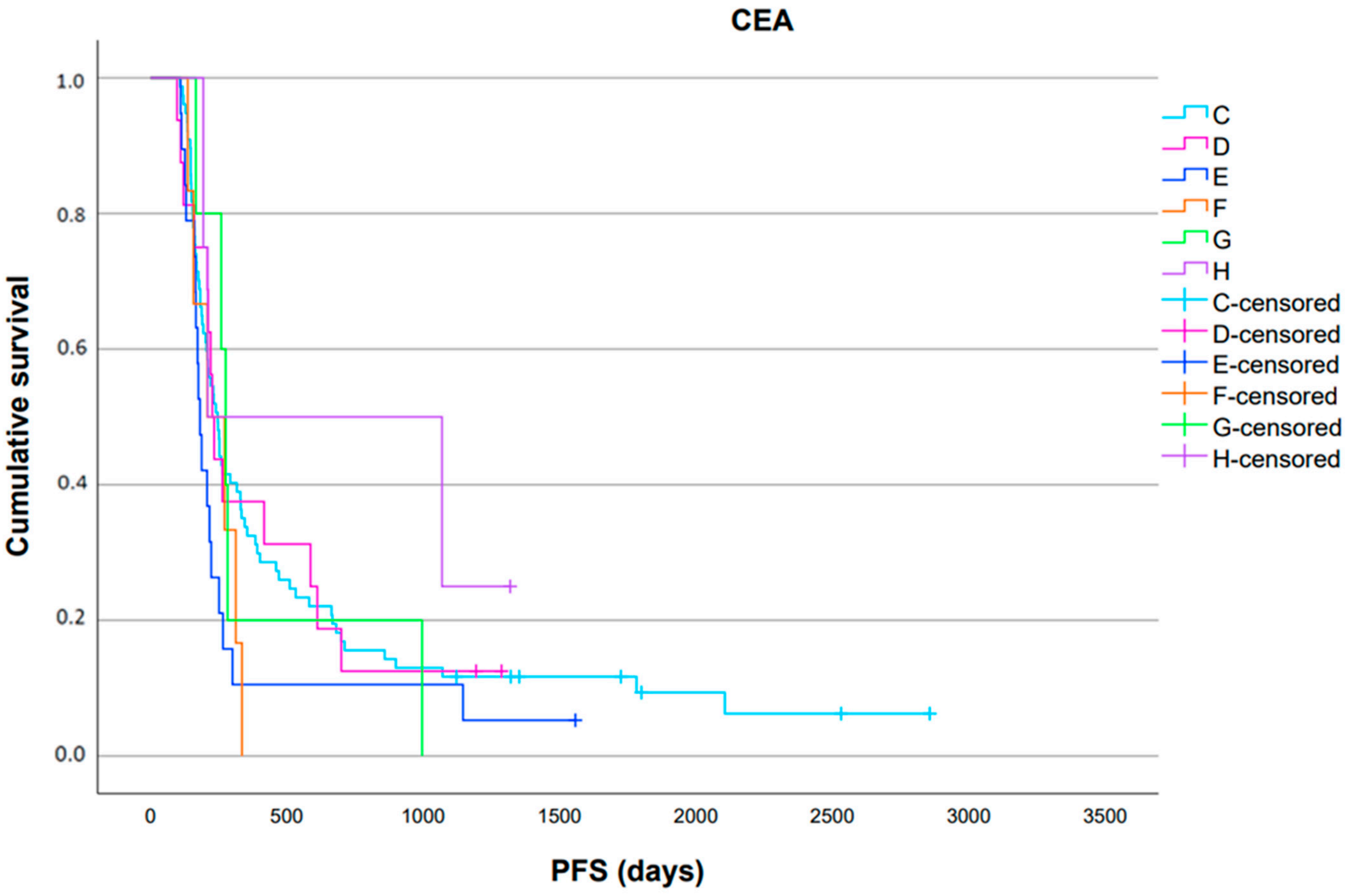

No. at risk								
	**0**	**500**	**1000**	**1500**	**2000**	**2500**	**3000**	**3500**
**C**	77 (0)	20 (0)	10 (0)	6 (3)	3 (5)	2 (5)	0 (7)	0 (7)
**D**	16 (0)	5 (0)	2 (0)	0 (2)	0 (2)	0 (2)	0 (2)	0 (2)
**E**	19 (0)	2 (0)	2 (0)	1 (0)	0 (1)	0 (1)	0 (1)	0 (1)
**F**	6 (0)	0 (0)	0 (0)	0 (0)	0 (0)	0 (0)	0 (0)	0 (0)
**G**	5 (0)	1 (0)	0 (0)	0 (0)	0 (0)	0 (0)	0 (0)	0 (0)
**H**	4 (0)	2 (0)	2 (0)	0 (1)	0 (1)	0 (1)	0 (1)	0 (1)

Figure S9. Progression-free survival associated with the STM dynamics subclassified as SeS for CEA. Abbreviations: STM, serum tumor marker; CEA, CarcinoEmbryonic Antigen; SeS, serological stable/unknown significance; PFS, progression-free survival; No., number.

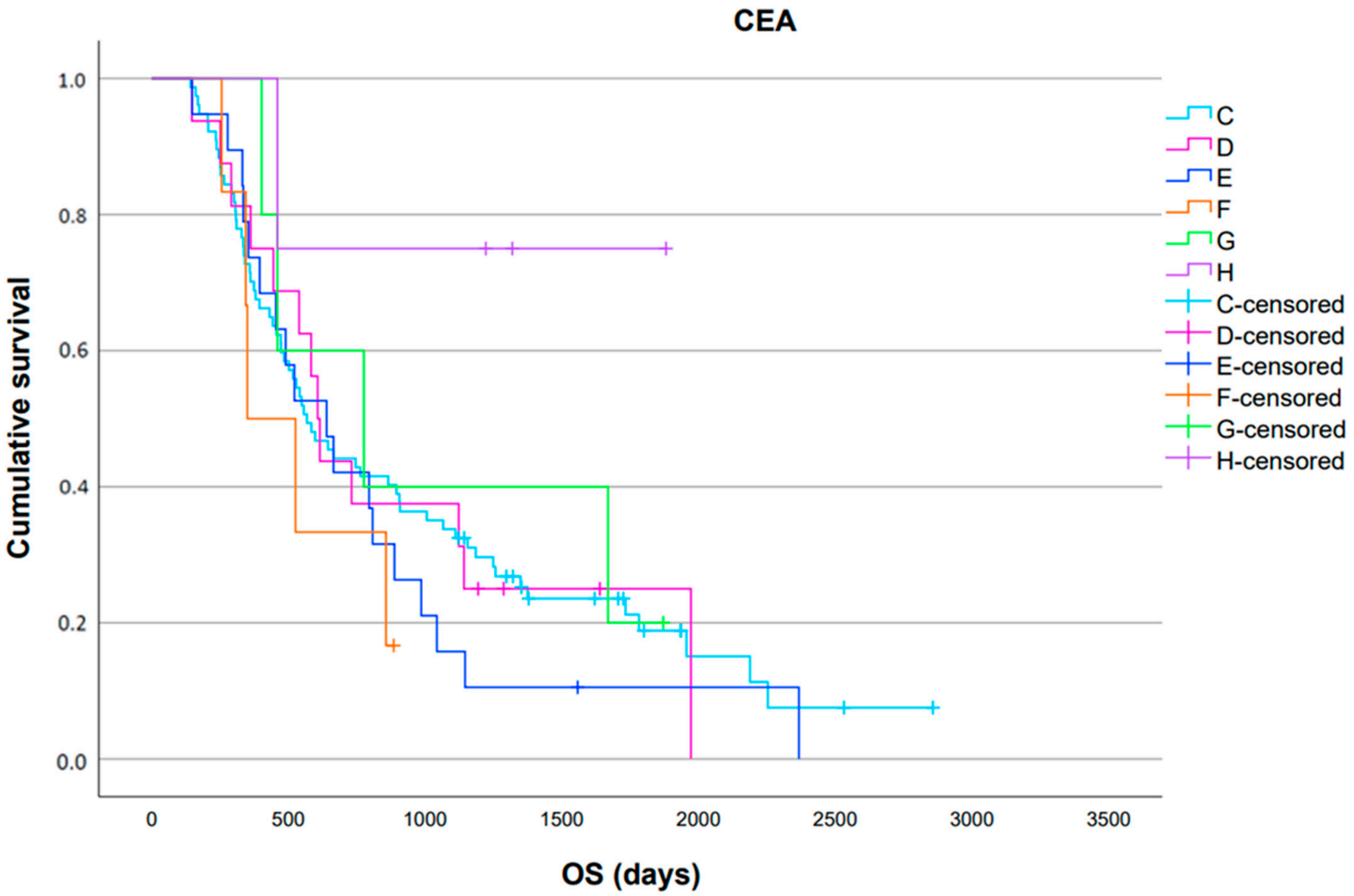

No. at risk								
	**0**	**500**	**1000**	**1500**	**2000**	**2500**	**3000**	**3500**
**C**	77 (0)	45 (0)	28 (0)	13 (6)	4 (12)	2 (12)	0 (14)	0 (14)
**D**	16 (0)	11 (0)	6 (0)	2 (2)	0 (3)	0 (3)	0 (3)	0 (3)
**E**	19 (0)	11 (0)	4 (0)	2 (0)	1 (1)	0 (1)	0 (1)	0 (1)
**F**	6 (0)	3 (0)	0 (1)	0 (1)	0 (1)	0 (1)	0 (1)	0 (1)
**G**	5 (0)	3 (0)	2 (0)	2 (0)	0 (1)	0 (1)	0 (1)	0 (1)
**H**	4 (0)	3 (0)	3 (0)	1 (2)	0 (3)	0 (3)	0 (3)	0 (3)

Figure S10. Overall survival associated with the STM dynamics subclassified as SeS for CEA. Abbreviations: STM, serum tumor marker; CEA, CarcinoEmbryonic Antigen; SeS, serological stable/unknown significance; OS, overall survival; No., number. .

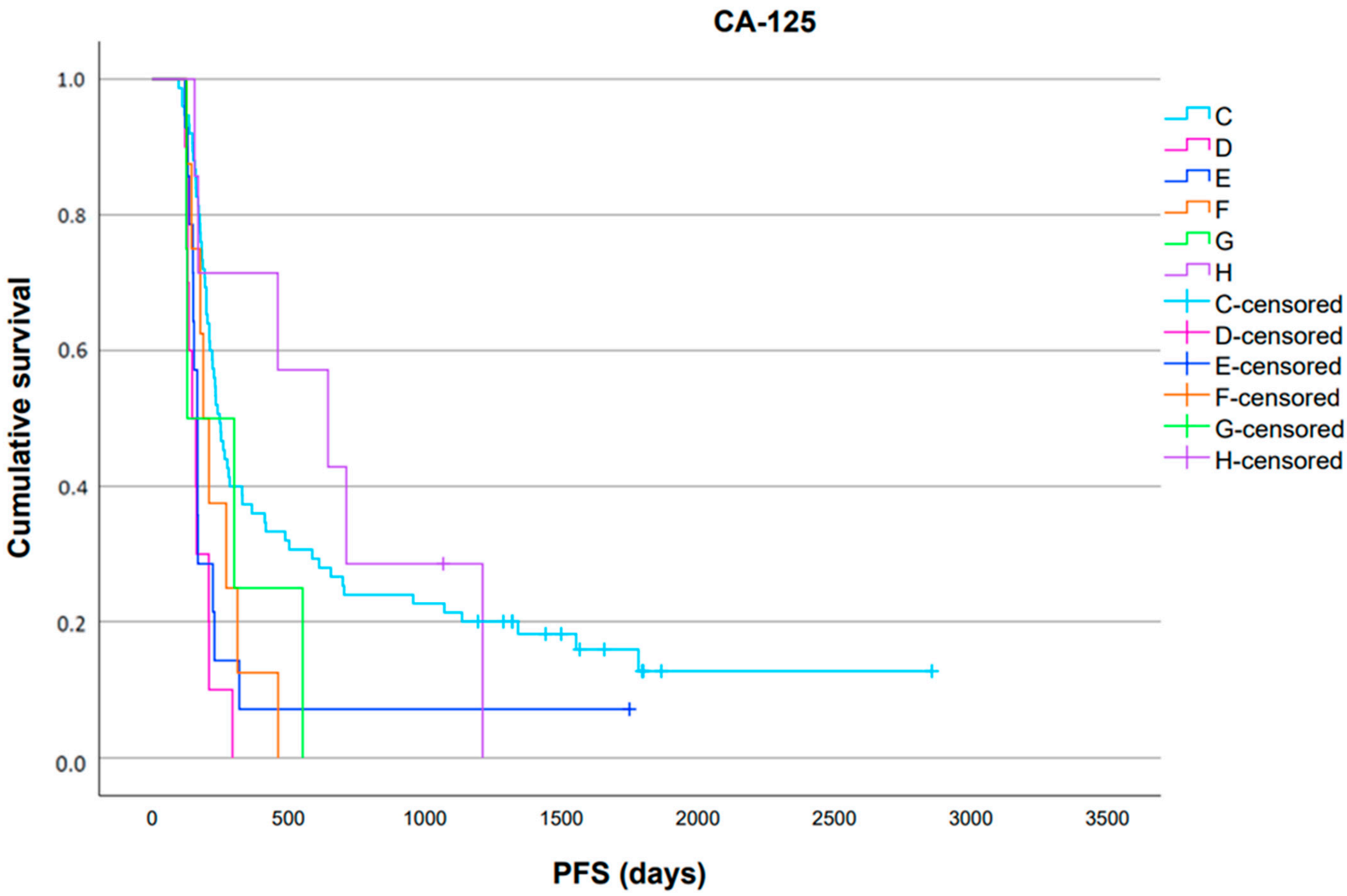

No. at risk								
	**0**	**500**	**1000**	**1500**	**2000**	**2500**	**3000**	**3500**
**C**	75 (0)	24 (0)	17 (0)	8 (6)	1 (11)	1 (11)	0 (12)	0 (12)
**D**	10 (0)	0 (0)	0 (0)	0 (0)	0 (0)	0 (0)	0 (0)	0 (0)
**E**	14 (0)	1 (0)	1 (0)	1 (0)	0 (1)	0 (1)	0 (1)	0 (1)
**F**	8 (0)	0 (0)	0 (0)	0 (0)	0 (0)	0 (0)	0 (0)	0 (0)
**G**	4 (0)	1 (0)	0 (0)	0 (0)	0 (0)	0 (0)	0 (0)	0 (0)
**H**	7 (0)	4 (0)	2 (0)	0 (1)	0 (1)	0 (1)	0 (1)	0 (1)

Figure S11. Progression-free survival associated with the STM dynamics subclassified as SeS for CA-125. Abbreviations: STM, serum tumor marker; CA-125, Cancer Antigen-125; SeS, serological stable/unknown significance; PFS, progression-free survival; No., number.

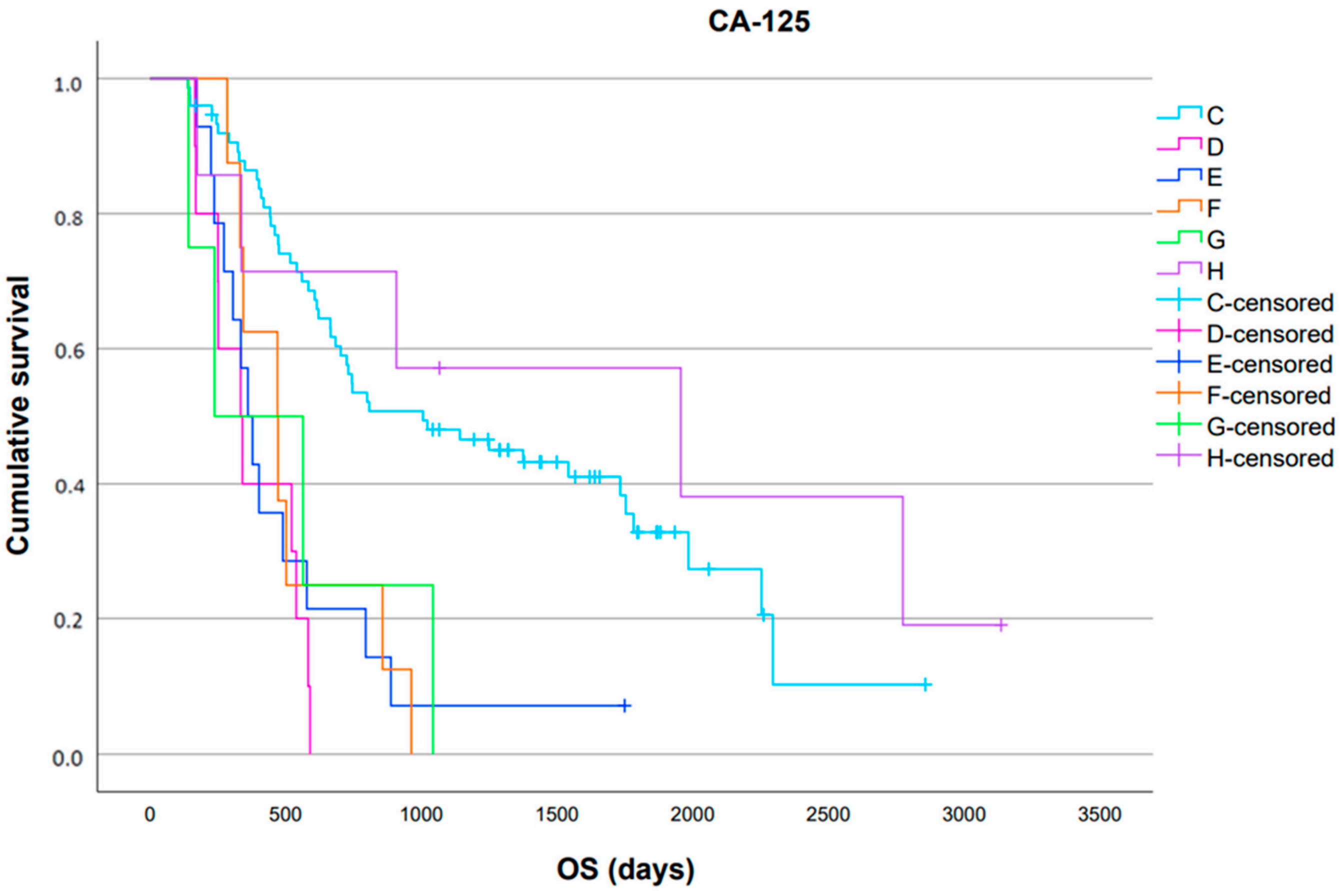

No. at risk								
	0	500	1000	1500	2000	2500	3000	3500
C	75 (0)	54 (2)	37 (2)	20 (14)	5 (24)	1 (26)	0 (27)	0 (27)
D	10 (0)	4 (0)	0 (0)	0 (0)	0 (0)	0 (0)	0 (0)	0 (0)
E	14 (0)	4 (0)	1 (0)	1 (0)	0 (1)	0 (1)	0 (1)	0 (1)
F	8 (0)	3 (0)	0 (0)	0 (0)	0 (0)	0 (0)	0 (0)	0 (0)
G	4 (0)	2 (0)	1 (0)	0 (0)	0 (0)	0 (0)	0 (0)	0 (0)
H	7 (0)	5 (0)	4 (0)	3 (1)	2 (1)	2 (1)	1 (1)	0 (2)

Figure S12. Overall survival associated with the STM dynamics subclassified as SeS for CA-125. Abbreviations: STM, serum tumor marker; CA-125, Cancer Antigen-125; SeS, serological stable/unknown significance; OS, overall survival; No., number.

Table S1. Overview of the possible individual serological response classification combinations and their corresponding combined serological response classification.

Individual serological response classification			Combined serological response classification
STM 1	STM 2	STM 3	
SeR	SeR	SeR	SeR
SeR	SeR	SeS	SeS
SeR	SeS	SeS	SeS
SeS	SeS	SeS	SeS
SeS	SeS	SeP	SeP
SeS	SeP	SeP	SeP
SeP	SeP	SeP	SeP
SeR	SeS	SeP	SeP
SeR	SeR	SeP	SeP
SeR	SeP	SeP	SeP

Abbreviations: STM, serum tumor marker; SeR, serological remission; SeS, serological stable/unknown significance; SeP, serological progression.

Table S2. Patient characteristics of the patient population at baseline according to the individualserological response classification for Cyfra 21.1.

	Patients, *n* = 256 (100.0%)			
	Serological remission [a], *n* = 28 (10.9%)	Serological stable/unknown significance [b], *n* = 117 (45.7%)	Serological progression [c], *n* = 111 (43.4%)	*p*-value
**General Characteristics**				
Age, mean (SD)	60.9 (10.4)	63.9 (9.4)	63.7 (9.5)	0.31
Gender (male), No. (%)	13 (46.4)	58 (49.6)	55 (49.5)	0.97
BMI, median (IQR)	25.9 (23.9–30.0)	24.3 (22.0–26.9)	24.2 (21.7–27.5)	0.09
ECOG PS (≥ 2), No. (%)	6 (21.4)	9 (7.7)	10 (9.0)	0.11
Ethnicity (Caucasian), No. (%)	24 (85.7)	105 (92.9)	102 (95.3)	0.18
Haemoglobin level <6.0 mmol/L, No. (%)	0 (0.0)	10 (8.6)	5 (4.6)	0.22
Albumin level <35 g/L, No. (%)	7 (29.2)	39 (33.9)	45 (42.1)	0.34
Kidney function ≤60 mL/min/1.73 m^2, No. (%)	1 (3.8)	24 (20.5)	15 (13.8)	0.08
**Smoking**				
Smoking status, No. (%)				0.82
Never smoker	4 (15.4)	10 (8.8)	10 (9.5)	
Ex-smoker	16 (61.5)	80 (70.8)	72 (68.6)	
Current smoker	6 (23.1)	23 (20.4)	23 (21.9)	
Pack years, median (IQR)	23.3 (8.8–40.0)	30.0 (20.0–40.0)	30.0 (15.0–40.1)	0.47
**Comorbidities**				
COPD (yes), No. (%)	8 (28.6)	30 (25.6)	23 (20.7)	0.56
Diabetes mellitus (yes), No. (%)	2 (7.1)	18 (15.4)	12 (10.8)	0.43
Auto-immune disease (yes), No. (%)	1 (3.6)	7 (6.0)	11 (9.9)	0.45
Kidney disease (yes), No. (%)	0 (0.0)	9 (7.7)	4 (3.6)	0.20
Liver disease (yes), No. (%)	0 (0.0)	1 (0.9)	1 (0.9)	>0.99
Cancer (yes), No. (%)	1 (3.6)	2 (1.7)	3 (2.7)	0.60

Metastasized (yes), No. (%)	1 (100.0)	2 (100.0)	1 (33.3)	0.60
**Medication use**				
Immunosuppressants, No. (%)	3 (11.1)	9 (7.8)	14 (12.7)	0.43
Aspirin/NSAIDs (yes), No. (%)	5 (18.5)	26 (22.6)	26 (23.6)	0.91
Anticoagulants (yes), No. (%)	4 (14.8)	26 (22.6)	23 (20.9)	0.74
Proton pump inhibitors (yes), *n* (%)	10 (37.0)	51 (44.3)	48 (43.6)	0.80
Antibiotics <3 months (yes), *n* (%)	3 (11.1)	13 (11.2)	11 (10.0)	0.96
**Tumor characteristics**				
Histology, No. (%)				0.38
Adenocarcinoma	17 (60.7)	90 (77.6)	83 (76.9)	
Squamous cell carcinoma	7 (25.0)	16 (13.8)	17 (15.7)	
Other	4 (14.3)	10 (8.6)	8 (7.4)	
PD-L1 expression, No. (%)				0.92
Negative (< 1%)	10 (38.5)	41 (42.7)	40 (44.0)	
Weak positive (1–49%)	7 (26.9)	23 (24.0)	18 (19.8)	
Strong positive (≥ 50%)	9 (34.6)	32 (33.3)	33 (36.3)	
Mutation status, No. (%)				
KRAS positive	4 (16.7)	45 (41.3)	35 (35.4)	0.07
EGFR positive	3 (12.5)	15 (13.8)	11 (11.0)	0.84
BRAF positive	2 (7.7)	10 (8.7)	5 (4.9)	0.51
ALK positive	0 (0.0)	1 (0.9)	1 (1.0)	>0.99
Lung cancer stage, No. (%)				0.43
Stage ≤III	3 (11.1)	6 (5.3)	9 (8.2)	
Localization of distant metastases, No. (%)				
Brain	6 (22.2)	24 (21.1)	18 (16.4)	0.61
Bone	9 (33.3)	25 (21.9)	36 (32.7)	0.16
Liver	2 (7.4)	12 (10.5)	10 (9.1)	0.95
Adrenal gland(s)	6 (22.2)	25 (21.9)	19 (17.3)	0.65
**Treatment**				
Current, No. (%)				0.91
ICI monotherapy [d]	12 (42.9)	54 (46.2)	48 (43.2)	
Dual ICI therapy [e]	0 (0.0)	0 (0.0)	1 (0.9)	
ICI and chemotherapy [f]	16 (57.1)	63 (53.8)	62 (55.9)	
Line of treatment, No. (%)				0.17
≥Second-line	13 (46.4)	62 (53.0)	45 (40.5)	
**Site of inclusion**				
RadboudUMC (yes), No. (%)	17 (60.7)	62 (53.0)	72 (64.9)	0.19

Abbreviations: Cyfra 21.1, Cytokeratin 19 fragment antigen; SD, standard deviation; No., number; BMI, body mass index; IQR, interquartile range; ECOG PS, Eastern Cooperative Oncology Group Performance Score; COPD, Chronic Obstructive Pulmonary Disease; NSAIDs, non-steroidal anti-inflammatory drugs; PD-L1, programmed death-ligand 1; KRAS, Kirsten rat sarcoma virus; EGFR, epidermal growth factor receptor; BRAF, B-Raf Proto-oncogene; ALK, anaplastic lymphoma kinase; ICI, immune checkpoint inhibitor; RadboudUMC, Radboud University Medical Centre. [a] Missing data: haemoglobin level ($n = 2$), albumin level ($n = 4$), kidney function ($n = 2$), smoking status ($n = 2$), pack years ($n = 3$), use of immunosuppressants ($n = 1$), use of aspirin/NSAIDs ($n = 1$), use of anticoagulants ($n = 1$), use of proton pump inhibitors ($n = 1$), recent use of antibiotics ($n = 1$), PD-L1 expression ($n = 2$), KRAS status ($n = 4$), EGFR status ($n = 4$), BRAF status ($n = 2$), ALK status ($n = 4$), lung cancer stage ($n = 1$), brain metastases ($n = 1$), bone metastases ($n = 1$), liver metastases ($n = 1$), adrenal gland(s) metastases ($n = 1$). [b] Missing data: BMI ($n = 1$), ethnicity ($n = 4$), haemoglobin level

(*n* = 1), albumin level (*n* = 2), smoking status (*n* = 4), pack years (*n* = 22), use of immunosuppressants (*n* = 1), use of aspirin/NSAIDs (*n* = 2), use of anticoagulants (*n* = 2), use of proton pump inhibitors (*n* = 2), recent use of antibiotics (*n* = 2), histology (*n* = 1), PD-L1 expression (*n* = 21), KRAS status (*n* = 9), EGFR status (*n* = 8), BRAF status (*n* = 2), ALK status (*n* = 10), lung cancer stage (*n* = 3), brain metastases (*n* = 3), bone metastases (*n* = 3), liver metastases (*n* = 3), adrenal gland(s) metastases (*n* = 3). [c] Missing data: ethnicity (*n* = 4), haemoglobin level (*n* = 2), albumin level (*n* = 4), kidney function (*n* = 2), smoking status (*n* = 6), pack years (*n* = 21), use of immunosuppressants (*n* = 1), use of aspirin/NSAIDs (*n* = 1), use of anticoagulants (*n* = 1), use of proton pump inhibitors (*n* = 1), recent use of antibiotics (*n* = 1), histology (*n* = 3), PD-L1 expression (*n* = 20), KRAS status (*n* = 12), EGFR status (*n* = 11), BRAF status (*n* = 9), ALK status (*n* = 14), lung cancer stage (*n* = 1), brain metastases (*n* = 1), bone metastases (*n* = 1), liver metastases (*n* = 1), adrenal gland(s) metastases (*n* = 1). [d] ICI monotherapy: nivolumab (*n* = 59), pembrolizumab (*n* = 48), atezolizumab (*n* = 5), durvalumab (*n* = 1), avelumab (*n* = 1). [e] Dual ICI therapy: nivolumab + ipilimumab (*n* = 1). [f] ICI and chemotherapy: pembrolizumab + cis-/carboplatin + pemetrexed (*n* = 88), pembrolizumab + cis-/carboplatin + paclitaxel (*n* = 22), atezolizumab + bevacizumab + carboplatin + paclitaxel (*n* = 30), nivolumab + carboplatin + gemcitabine (*n* = 1).

Table S3. Patient characteristics of the patient population at baseline according to the individual serological response classification for CEA.

	**Patients, *n* = 252 (98.4%)**			
	**Serological remission [a], *n* = 37 (14.7%)**	**Serological stable/unknown significance [b], *n* = 127 (50.4%)**	**Serological progression [c], *n* = 88 (34.9%)**	***p*-value**
**General Characteristics**				
Age, mean (SD)	62.4 (10.3)	63.9 (9.5)	63.7 (9.3)	0.71
Gender (male), No. (%)	15 (40.5)	60 (47.2)	49 (55.7)	0.27
BMI, median (IQR)	24.4 (22.2–27.8)	24.6 (22.6–27.5)	24.1 (21.3–27.6)	0.66
ECOG PS (≥ 2), No. (%)	4 (10.8)	14 (11.0)	6 (6.8)	0.56
Ethnicity (Caucasian), No. (%)	33 (91.7)	114 (91.9)	80 (95.2)	0.62
Haemoglobin level < 6.0 mmol/L, No. (%)	5 (13.5)	5 (4.0)	4 (4.7)	0.10
Albumin level < 35 g/L, No. (%)	12 (33.3)	48 (39.0)	30 (36.1)	0.81
Kidney function ≤ 60 mL/min/1.73 m^2, No. (%)	5 (13.5)	16 (12.7)	17 (20.0)	0.36
**Smoking**				
Smoking status, No. (%)				0.63
Never smoker	5 (14.3)	9 (7.6)	9 (10.3)	
Ex-smoker	21 (60.0)	86 (72.3)	59 (67.8)	
Current smoker	9 (25.7)	24 (20.2)	19 (21.8)	
Pack years, median (IQR)	24.1 (8.8–40.0)	30.0 (20.0–40.5)	28.0 (10.0–40.0)	0.22
**Comorbidities**				
COPD (yes), No. (%)	7 (18.9)	38 (29.9)	16 (18.2)	0.11
Diabetes mellitus (yes), No. (%)	4 (10.8)	16 (12.6)	12 (13.6)	0.93
Auto-immune disease (yes), No. (%)	1 (2.7)	9 (7.1)	9 (10.2)	0.37
Kidney disease (yes), No. (%)	2 (5.4)	6 (4.7)	5 (5.7)	0.93
Liver disease (yes), No. (%)	0 (0.0)	2 (1.6)	0 (0.0)	0.65
Cancer (yes), No. (%)	0 (0.0)	5 (3.9)	1 (1.1)	0.43
Metastasized (yes), No. (%)	0 (0.0)	4 (80.0)	0 (0.0)	0.33
**Medication use**				

Immunosuppressants, No. (%)	0 (0.0)	15 (11.9)	10 (11.5)	0.06
Aspirin/NSAIDs (yes), No. (%)	8 (22.2)	30 (23.8)	17 (19.8)	0.80
Anticoagulants (yes), No. (%)	8 (22.2)	29 (23.0)	16 (18.6)	0.72
Proton pump inhibitors (yes), *n* (%)	13 (36.1)	63 (50.0)	31 (36.0)	0.09
Antibiotics <3 months (yes), *n* (%)	2 (5.6)	17 (13.5)	7 (8.1)	0.32
**Tumor characteristics**				
Histology, No. (%)				0.02 *
Adenocarcinoma	27 (73.0)	87 (69.0)	75 (88.2)	
Squamous cell carcinoma	7 (18.9)	26 (20.6)	5 (5.9)	
Other	3 (8.1)	13 (10.3)	5 (5.9)	
PD-L1 expression, No. (%)				0.73
Negative (< 1%)	15 (48.4)	43 (39.8)	33 (45.2)	
Weak positive (1–49%)	7 (22.6)	23 (21.3)	18 (24.7)	
Strong positive (≥ 50%)	9 (29.0)	42 (38.9)	22 (30.1)	
Mutation status, No. (%)				
KRAS positive	10 (31.3)	45 (40.2)	28 (33.3)	0.51
EGFR positive	7 (21.2)	9 (8.0)	13 (15.5)	0.07
BRAF positive	4 (10.8)	7 (5.7)	6 (9.8)	0.49
ALK positive	1 (3.1)	0 (0.0)	1 (1.2)	0.12
Lung cancer stage, No. (%)				0.60
Stage ≤ III	3 (8.1)	7 (5.7)	8 (9.2)	
Localization of distant metastases, No. (%)				
Brain	7 (18.9)	23 (18.7)	18 (20.7)	0.95
Bone	9 (24.3)	33 (26.8)	28 (32.2)	0.62
Liver	8 (21.6)	8 (6.5)	8 (9.2)	0.03 *
Adrenal gland(s)	7 (18.9)	26 (21.1)	16 (18.4)	0.93
**Treatment**				
Current, No. (%)				0.11
ICI monotherapy [d]	10 (27.0)	61 (48.0)	39 (44.3)	
Dual ICI therapy [e]	0 (0.0)	1 (0.8)	0 (0.0)	
ICI and chemotherapy [f]	27 (73.0)	65 (51.2)	49 (55.7)	
Line of treatment, No. (%)				0.52
≥ Second-line	17 (45.9)	55 (43.3)	45 (51.1)	
**Site of inclusion**				
RadboudUMC (yes), No. (%)	25 (67.6)	75 (59.1)	51 (58.0)	0.61

* *p*-value < 0.05 when comparing the three serological response patterns. Abbreviations: CEA, CarcinoEmbryonic Antigen; SD, standard deviation; No., number; BMI, body mass index; IQR, interquartile range; ECOG PS, Eastern Cooperative Oncology Group Performance Score; COPD, Chronic Obstructive Pulmonary Disease; NSAIDs, non-steroidal anti-inflammatory drugs; PD-L1, programmed death-ligand 1; KRAS, Kirsten rat sarcoma virus; EGFR, epidermal growth factor receptor; BRAF, B-Raf Proto-oncogene; ALK, anaplastic lymphoma kinase; ICI, immune checkpoint inhibitor; RadboudUMC, Radboud University Medical Centre. [a] Missing data: ethnicity (*n* = 1), albumin level (*n* = 1), smoking status (*n* = 2), pack years (*n* = 7), use of immunosuppressants (*n* = 1), use of aspirin/NSAIDs (*n* = 1), use of anticoagulants (*n* = 1), use of proton pump inhibitors (*n* = 1), recent use of antibiotics (*n* = 1), PD-L1 expression (*n* = 6), KRAS status (*n* = 5), EGFR status (*n* = 4), ALK status (*n* = 5). [b] Missing data: BMI (*n* = 1), ethnicity (*n* = 3), haemoglobin level (*n* = 2), albumin level (*n* = 4), kidney function (*n* = 1), smoking status (*n* = 8), pack years (*n* = 21), use of immunosuppressants (*n* = 1), use of aspirin/NSAIDs (*n* = 1), use of anticoagulants (*n* = 1), use of proton pump inhibitors (*n* = 1), recent use of antibiotics (*n* = 1), histology (*n* = 1), PD-L1 expression (*n* = 19), KRAS status (*n* = 15), EGFR status (*n* = 15), BRAF

status ($n = 5$), ALK status ($n = 15$), lung cancer stage ($n = 4$), brain metastases ($n = 4$), bone metastases ($n = 4$), liver metastases ($n = 4$), adrenal gland(s) metastases ($n = 4$). [c] Missing data: ethnicity ($n = 4$), haemoglobin level ($n = 3$), albumin level ($n = 5$), kidney function ($n = 3$), smoking status ($n = 1$), pack years ($n = 16$), use of immunosuppressants ($n = 1$), use of aspirin/NSAIDs ($n = 2$), use of anticoagulants ($n = 2$), use of proton pump inhibitors ($n = 2$), recent use of antibiotics ($n = 2$), histology ($n = 3$), PD-L1 expression ($n = 15$), KRAS status ($n = 4$), EGFR status ($n = 4$), BRAF status ($n = 6$), ALK status ($n = 7$), lung cancer stage ($n = 1$), brain metastases ($n = 1$), bone metastases ($n = 1$), liver metastases ($n = 1$), adrenal gland(s) metastases ($n = 1$). [d] ICI monotherapy: nivolumab ($n = 55$), pembrolizumab ($n = 48$), atezolizumab ($n = 5$), durvalumab ($n = 1$), avelumab ($n = 1$). [e] Dual ICI therapy: nivolumab + ipilimumab ($n = 1$). [f] ICI and chemotherapy: pembrolizumab + cis-/carboplatin + pemetrexed ($n = 88$), pembrolizumab + cis-/carboplatin + paclitaxel ($n = 22$), atezolizumab + bevacizumab + carboplatin + paclitaxel ($n = 30$), nivolumab + carboplatin + gemcitabine ($n = 1$).

Table S4. Patient characteristics of the patient population at baseline according to the individual serological response classification for CA-125.

	**Patients, *n* = 239 (93.4%)**			
	**Serological remission [a], *n* = 57 (23.8%)**	**Serological stable/unknown significance [b], *n* = 118 (49.4%)**	**Serological progression [c], *n* = 64 (26.8%)**	***p*-value**
**General Characteristics**				
Age, mean (SD)	63.0 (8.6)	63.5 (9.7)	62.2 (10.0)	0.69
Gender (male), No. (%)	25 (43.9)	60 (50.8)	33 (51.6)	0.66
BMI, median (IQR)	23.9 (21.1–26.2)	24.9 (22.8–28.1)	23.7 (21.5–27.7)	0.06
ECOG PS (≥ 2), No. (%)	9 (15.8)	6 (5.9)	10 (15.6)	0.02 *
Ethnicity (Caucasian), No. (%)	5 (8.8)	5 (4.4)	7 (11.5)	0.19
Haemoglobin level < 6.0 mmol/L, No. (%)	4 (7.1)	6 (5.2)	4 (6.5)	0.82
Albumin level < 35 g/L, No. (%)	14 (25.5)	37 (32.2)	28 (47.5)	0.04 *
Kidney function ≤ 60 mL/min/1.73 m^2, No. (%)	8 (14.3)	20 (17.1)	11 (17.7)	0.90
**Smoking**				
Smoking status, No. (%)				0.13
Never smoker	9 (16.1)	10 (9.1)	4 (6.6)	
Ex-smoker	34 (60.7)	73 (66.4)	49 (80.3)	
Current smoker	13 (23.2)	27 (24.5)	8 (13.1)	
Pack years, median (IQR)	25.0 (10.0–40.0)	30.0 (17.0–40.0)	25.5 (20.0–40.4)	0.51
**Comorbidities**				
COPD (yes), No. (%)	16 (28.1)	29 (24.6)	11 (17.2)	0.33
Diabetes mellitus (yes), No. (%)	6 (10.5)	16 (13.6)	6 (9.4)	0.73
Auto-immune disease (yes), No. (%)	1 (1.8)	11 (9.3)	6 (9.4)	0.15
Kidney disease (yes), No. (%)	1 (1.8)	10 (8.5)	2 (3.1)	0.15
Liver disease (yes), No. (%)	1 (1.8)	0 (0.0)	1 (1.6)	0.26
Cancer (yes), No. (%)	2 (3.5)	2 (1.7)	1 (1.6)	0.72
Metastasized (yes), No. (%)	2 (100.0)	1 (50.0)	0 (0.0)	0.60
**Medication use**				
Immunosuppressants, No. (%)	5 (8.8)	12 (10.4)	8 (12.7)	0.82
Aspirin/NSAIDs (yes), No. (%)	12 (21.1)	27 (23.5)	17 (27.0)	0.77
Anticoagulants (yes), No. (%)	9 (15.8)	26 (22.6)	13 (20.6)	0.60
Proton pump inhibitors (yes), *n* (%)	26 (45.6)	51 (44.3)	26 (41.3)	0.89
Antibiotics <3 months (yes), *n* (%)	9 (15.8)	10 (8.7)	7 (11.1)	0.36

**Tumor characteristics**				
Histology, No. (%)				0.86
Adenocarcinoma	42 (75.0)	85 (73.3)	49 (79.0)	
Squamous cell carcinoma	11 (19.6)	19 (16.4)	8 (12.9)	
Other	4 (7.1)	12 (10.3)	5 (8.1)	
PD-L1 expression, No. (%)				0.55
Negative (< 1%)	22 (45.8)	39 (33.6)	21 (44.7)	
Weak positive (1–49%)	14 (29.2)	23 (19.8)	10 (21.3)	
Strong positive (≥ 50%)	12 (25.0)	39 (33.6)	16 (34.0)	
Mutation status, No. (%)				
KRAS positive	16 (31.4)	43 (41.3)	20 (33.3)	0.39
EGFR positive	13 (25.5)	10 (9.4)	5 (8.3)	0.02 *
BRAF positive	3 (5.4)	7 (6.3)	4 (6.8)	>0.99
ALK positive	1 (2.0)	0 (0.0)	0 (0.0)	0.24
Lung cancer stage, No. (%)				0.63
Stage ≤ III	3 (5.4)	10 (8.7)	3 (4.8)	
Localization of distant metastases, No. (%)				
Brain	14 (25.0)	23 (20.0)	10 (15.9)	0.48
Bone	23 (41.1)	21 (18.3)	20 (31.7)	0.005 *
Liver	8 (14.3)	10 (8.7)	4 (6.3)	0.35
Adrenal gland(s)	12 (21.4)	21 (18.3)	15 (23.8)	0.66
**Treatment**				
Current, No. (%)				0.17
ICI monotherapy [d]	21 (36.8)	57 (48.3)	33 (51.6)	
Dual ICI therapy [e]	0 (0.0)	0 (0.0)	1 (1.6)	
ICI and chemotherapy [f]	36 (63.2)	61 (51.7)	30 (46.9)	
Line of treatment, No. (%)				0.08
≥ Second-line	35 (61.4)	52 (44.1)	29 (45.3)	
**Site of inclusion**				
RadboudUMC (yes), No. (%)	27 (47.4)	70 (59.3)	37 (57.8)	0.32

* p-value < 0.05 when comparing the three serological response patterns. Abbreviations: CA-125, Cancer Antigen-125; IQR, interquartile range; No., number; BMI, body mass index; ECOG PS, Eastern Cooperative Oncology Group Performance Score; COPD, Chronic Obstructive Pulmonary Disease; NSAIDs, non-steroidal anti-inflammatory drugs; PD-L1, programmed death-ligand 1; KRAS, Kirsten rat sarcoma virus; EGFR, epidermal growth factor receptor; BRAF, B-Raf Proto-oncogene; ALK, anaplastic lymphoma kinase; ICI, immune checkpoint inhibitor; RadboudUMC, Radboud University Medical Centre. [a] Missing data: haemoglobin level (n = 1), albumin level (n = 2), kidney function (n = 1), smoking status (n = 1), pack years (n = 9), PD-L1 expression (n = 9), KRAS status (n = 6), EGFR status (n = 6), BRAF status (n = 1), ALK status (n = 6), lung cancer stage (n = 1), brain metastases (n = 1), bone metastases (n = 1), liver metastases (n = 1), adrenal gland(s) metastases (n = 1).[b] Missing data: ethnicity (n = 5), haemoglobin level (n = 2), albumin level (n = 3), kidney function (n = 1), smoking status (n = 8), pack years (n = 19), use of immunosuppressants (n = 3), use of aspirin/NSAIDs (n = 3), use of anticoagulants (n = 3), use of proton pump inhibitors (n = 3), recent use of antibiotics (n = 3), histology (n = 2), PD-L1 expression (n = 17), KRAS status (n = 14), EGFR status (n = 12), BRAF status (n = 6), ALK status (n = 14), lung cancer stage (n = 3), brain metastases (n = 3), bone metastases (n = 3), liver metastases (n = 3), adrenal gland(s) metastases (n = 3). [c] Missing data: BMI (n = 1), ethnicity (n = 3), haemoglobin level (n = 2), albumin level (n = 5), kidney function (n = 2), smoking status (n = 3), pack years (n = 16), use of aspirin/NSAIDs (n = 1), use of anticoagulants (n = 1), use of proton pump inhibitors (n = 1), recent use of antibiotics (n = 1), histology (n = 2), PD-

L1 expression (n = 17), KRAS status (n = 4), EGFR status (n = 4), BRAF status (n = 5), ALK status (n = 7), lung cancer stage (n = 1), brain metastases (n = 1), bone metastases (n = 1), liver metastases (n = 1), adrenal gland(s) metastases (n = 1). [d] ICI monotherapy: nivolumab (n = 59), pembrolizumab (n = 46), atezolizumab (n = 5), durvalumab (n = 1), avelumab (n = 1). [e] Dual ICI therapy: nivolumab + ipilimumab (n = 1). [f] ICI and chemotherapy: pembrolizumab + cis-/carboplatin + pemetrexed (n = 77), pembrolizumab + cis-/carboplatin + paclitaxel (n = 20), atezolizumab + bevacizumab + carboplatin + paclitaxel (n = 28), nivolumab + carboplatin + gemcitabine (n = 1).

Table S5. Patient characteristics of the patient population at baseline according to the combined serological response classification.

	**Patients, n = 256 (100.0%)**			
	**Serological remission [a], n = 6 (2.3%)**	**Serological stable/unknown significance [b], n = 89 (34.8%)**	**Serological progression [c], n = 161 (62.9%)**	***p*-value**
**General Characteristics**				
Age, mean (SD)	63.3 (11.6)	63.4 (9.4)	63.6 (9.6)	0.99
Gender (male), No. (%)	3 (50.0)	39 (43.8)	84 (52.2)	0.43
BMI, median (IQR)	24.6 (22.1–27.5)	24.7 (22.5–27.0)	24.2 (21.7–27.8)	0.91
ECOG PS (≥ 2), No. (%)	2 (33.3)	8 (9.0)	15 (9.3)	0.16
Ethnicity (Caucasian), No. (%)	1 (16.7)	5 (5.7)	11 (7.1)	0.41
Haemoglobin level <6.0 mmol/L, No. (%)	0 (0.0)	6 (6.9)	9 (5.7)	0.85
Albumin level < 35 g/L, No. (%)	3 (50.0)	28 (32.9)	60 (38.7)	0.49
Kidney function ≤ 60 mL/min/1.73 m^2, No. (%)	1 (16.7)	12 (13.6)	27 (17.1)	0.73
**Smoking**				
Smoking status, No. (%)				0.50
Never smoker	1 (16.7)	10 (11.9)	13 (8.4)	
Ex-smoker	4 (66.7)	53 (63.1)	111 (72.1)	
Current smoker	1 (16.7)	21 (25.0)	30 (19.5)	
Pack years, median (IQR)	19.3 (3.8–27.4)	30.0 (15.5–40.0)	30.0 (16.3–41.6)	0.27
**Comorbidities**				
COPD (yes), No. (%)	2 (33.3)	24 (27.0)	35 (21.7)	0.50
Diabetes mellitus (yes), No. (%)	0 (0.0)	14 (15.7)	18 (11.1)	0.43
Auto-immune disease (yes), No. (%)	0 (0.0)	6 (6.7)	13 (8.1)	0.88
Kidney disease (yes), No. (%)	0 (0.0)	6 (6.7)	7 (4.3)	0.67
Liver disease (yes), No. (%)	0 (0.0)	1 (1.1)	1 (0.6)	>0.99
Cancer (yes), No. (%)	0 (0.0)	3 (3.4)	3 (1.9)	0.71
Metastasized (yes), No. (%)	0 (0.0)	3 (100.0)	1 (33.3)	0.40
**Medication use**				
Immunosuppressants, No. (%)	0 (0.0)	7 (8.0)	19 (11.9)	0.56
Aspirin/NSAIDs (yes), No. (%)	1 (16.7)	20 (22.7)	36 (22.8)	>0.99
Anticoagulants (yes), No. (%)	0 (0.0)	18 (20.5)	35 (22.2)	0.61
Proton pump inhibitors (yes), n (%)	2 (33.3)	42 (47.7)	65 (41.1)	0.61
Antibiotics <3 months (yes), n (%)	0 (0.0)	9 (10.2)	18 (11.4)	0.92
**Tumor characteristics**				
Histology, No. (%)				0.39
Adenocarcinoma	4 (66.7)	62 (70.5)	124 (78.5)	
Squamous cell carcinoma	2 (33.3)	17 (19.3)	21 (13.3)	

Other	0 (0.0)	9 (10.2)	13 (8.2)	
PD-L1 expression, No. (%)				0.29
Negative (< 1%)	4 (66.7)	28 (38.9)	59 (43.7)	
Weak positive (1–49%)	2 (33.3)	19 (26.4)	27 (20.0)	
Strong positive (≥ 50%)	0 (0.0)	25 (34.7)	49 (36.3)	
Mutation status, No. (%)				
KRAS positive	1 (20.0)	33 (40.7)	50 (34.5)	0.54
EGFR positive	1 (20.0)	11 (13.4)	17 (11.6)	0.58
BRAF positive	0 (0.0)	8 (9.0)	9 (6.1)	0.64
ALK positive	0 (0.0)	1 (1.2)	1 (0.7)	>0.99
Lung cancer stage, No. (%)				0.27
Stage ≤ III	1 (16.7)	4 (4.7)	13 (8.2)	
Localization of distant metastases, No. (%)				
Brain	1 (16.7)	20 (23.3)	27 (17.0)	0.44
Bone	1 (16.7)	24 (27.9)	45 (28.3)	>0.99
Liver	1 (16.7)	10 (11.6)	13 (8.2)	0.33
Adrenal gland(s)	1 (16.7)	18 (20.9)	31 (19.5)	0.95
**Treatment**				
Current, No. (%)				0.12
ICI monotherapy [d]	0 (0.0)	40 (44.9)	74 (46.0)	
Dual ICI therapy [e]	0 (0.0)	0 (0.0)	1 (0.6)	
ICI and chemotherapy [f]	6 (100.0)	49 (55.1)	86 (53.4)	
Line of treatment, No. (%)				0.74
≥ Second-line	2 (33.3)	44 (49.4)	74 (46.0)	
**Site of inclusion**				
RadboudUMC (yes), No. (%)	5 (83.3)	48 (53.9)	98 (60.9)	0.28

Abbreviations: SD, standard deviation; No., number; BMI, body mass index; IQR, interquartile range; ECOG PS, Eastern Cooperative Oncology Group Performance Score; COPD, Chronic Obstructive Pulmonary Disease; NSAIDs, non-steroidal anti-inflammatory drugs; PD-L1, programmed death-ligand 1; KRAS, Kirsten rat sarcoma virus; EGFR, epidermal growth factor receptor; BRAF, B-Raf Proto-oncogene; ALK, anaplastic lymphoma kinase; ICI, immune checkpoint inhibitor; RadboudUMC, Radboud University Medical Centre.[a] Missing data: KRAS status (n = 1), EGFR status (n = 1), ALK status (n = 1). [b] Missing data: ethnicity (n = 1), haemoglobin level (n = 2), albumin level (n = 4), kidney function (n = 1), smoking status (n = 5), pack years (n = 17), use of immunosuppressants (n = 1), use of aspirin/NSAIDs (n = 1), use of anticoagulants (n = 1),use of proton pump inhibitors (n = 1), recent use of antibiotics (n = 1), histology (n = 1), PD-L1 expression (n = 17), KRAS status (n = 8), EGFR status (n = 7), ALK status (n = 8), lung cancer stage (n = 3), brain metastases (n = 3), bone metastases (n = 3), liver metastases (n = 3), adrenal gland(s) metastases (n = 3). [c] Missing data: BMI (n = 1), ethnicity (n = 7), haemoglobin level (n = 3), albumin level (n = 6), kidney function (n = 3), smoking status (n = 7), pack years (n = 29), use of immunosuppressants (n = 2), use of aspirin/NSAIDs (n = 3), use of anticoagulants (n = 3), use of proton pump inhibitors (n = 3), recent use of antibiotics (n = 3), histology (n = 3), PD-L1 expression (n = 26), KRAS status (n = 16), EGFR status (n = 15), BRAF status (n = 13), ALK status (n = 19), lung cancer stage (n = 2), brain metastases (n = 2), bone metastases (n = 2), liver metastases (n = 2), adrenal gland(s) metastases (n = 2). [d] ICI monotherapy: nivolumab (n = 59), pembrolizumab (n = 48), atezolizumab (n = 5), durvalumab (n = 1), avelumab (n = 1). [e] Dual ICI therapy: nivolumab + ipilimumab (n = 1). [f] ICI and chemotherapy: pembrolizumab + cis-/carboplatin + pemetrexed (n = 88), pembrolizumab + cis-/carboplatin + paclitaxel (n = 22), atezolizumab + bevacizumab + carboplatin + paclitaxel (n = 30), nivolumab + carboplatin + gemcitabine (n = 1).

Table S6. Overview of the proportion of the 12 distinct recurring dynamics and corresponding serological response patterns for the individual and combined serological response classifications across advanced-stage NSCLC patients with an initial response to ICI-containing treatment.

	**Serological response pattern**	**STM dynamic**
**Individual serological response classification - Cyfra 21.1 (*n* = 256)**	SeR (*n* = 28, 10.9%)	A (*n* = 10, 3.9%)
		B (*n* = 18, 7.0%)
	SeS (*n* = 117, 45.7%)	C (*n* = 12, 4.7%)
		D (*n* = 22, 8.6%)
		E (*n* = 22, 8.6%)
		F (*n* = 16, 6.3%)
		G (*n* = 22, 8.6%)
		H (*n* = 23, 9.0%)
	SeP (*n* = 111, 43.4%)	I (*n* = 9, 3.5%)
		J (*n* = 31, 12.1%)
		K (*n* = 22, 8.6%)
		L (*n* = 49, 19.1%)
**Individual serological response classification - CEA (*n* = 252)**	SeR (*n* = 37, 14.7%)	A (*n* = 8, 3.2%)
		B (*n* = 29, 11.5%)
	SeS (*n* = 127, 50.4%)	C (*n* = 77, 30.6%)
		D (*n* = 16, 6.3%)
		E (*n* = 19, 7.5%)
		F (*n* = 6, 2.4%)
		G (*n* = 5, 2.0%)
		H (*n* = 4, 1.6%)
	SeP (*n* = 88, 34.3%)	I (*n* = 10, 4.0%)
		J (*n* = 32, 12.7%)
		K (*n* = 11, 4.4%)
		L (*n* = 35, 13.9%)
**Individual serological response classification - CA-125 (*n* = 239)**	SeR (*n* = 57, 23.8%)	A (*n* = 11, 4.6%)
		B (*n* = 46, 19.2%)
	SeS (*n* = 118, 49.4%)	C (*n* = 75, 31.4%)
		D (*n* = 10, 4.2%)
		E (*n* = 14, 5.9%)
		F (*n* = 8, 3.3%)
		G (*n* = 4, 1.7%)
		H (*n* = 7, 2.9%)
	SeP (*n* = 64, 26.8%)	I (*n* = 16, 6.7%)
		J (*n* = 12, 5.0%)
		K (*n* = 18, 7.5%)
		L (*n* = 18, 7.5%)
**Combined serological response classification (*n* = 256)**	SeR (*n* = 6, 2.3%)	
	SeS (*n* = 89, 34.8%)	
	SeP (*n* = 161, 62.9%)	

Abbreviations: Cyfra 21.1, Cytokeratin 19 fragment antigen; CEA, CarcinoEmbryonic Antigen: CA-125, Cancer Antigen-125; NSCLC, non-small cell lung cancer; ICI, immune checkpoint inhibitor; STM, serum tumor marker; SeR, serological remission; SeS, serological stable/unknown significance; SeP, serological progression.

Table S7. Overview of the added value of the three main serological response patterns for the individual and combined serological response classifications in distinguishing patients who achieve a durable response versus patients who develop secondary treatment resistance.

	Durable response (*n* = 38) [a]		Secondary treatment resistance (*n* = 218) [b]		*p*-value
**Individual serological response classification - Cyfra 21.1**	SeR (*n* = 8, 7.9%)	A (*n* = 4, 10.5%)	SeR (*n* = 20, 9.2%)	A (*n* = 6, 2.8%)	<0.001 *
		B (*n* = 4, 10.5%)		B (*n* = 14, 6.4%)	
	SeS (*n* = 27, 71.1%)	C (*n* = 2, 5.3%)	SeS (*n* = 90, 41.3%)	C (*n* = 8, 3.7%)	
		D (*n* = 1, 2.6%)		D (*n* = 20, 9.2%	
		E (*n* = 1, 2.6%)		E (*n* = 21, 9.6%	
		F (*n* = 9, 23.7%)		F (*n* = 15, 6.9%	
		G (*n* = 10, 26.3%)		G (*n* = 13, 6.0%)	
		H (*n* = 2, 5.3%)		H (*n* = 13, 6.0%)	
	SeP (*n* = 3, 7.9%)	I (*n* = 1, 2.6%)	SeP (*n* = 108, 49.5%)	I (*n* = 7, 3.2%)	
		J (*n* = 0, 0.0%)		J (*n* = 30, 13.8%)	
		K (*n* = 0, 0.0%)		K (*n* = 22, 10.1%)	
		L (*n* = 2, 5.3%)		L (*n* = 49, 22.5%)	
**Individual serological response classification - CEA**	SeR (*n* = 12, 31.6%)	A (*n* = 3, 7.9%)	SeR (*n* = 25, 11.7%)	A (*n* = 5, 2.3%)	0.007 *
		B (*n* = 9, 23.7%)		B (*n* = 20, 9.3%)	
	SeS (*n* = 18, 47.4%)	C (*n* = 11, 28.9%)	SeS (*n* = 109, 50.9%)	C (*n* = 66, 30.8%)	
		D (*n* = 2, 5.3%)		D (*n* = 14, 6.5%)	
		E (*n* = 2, 5.3%)		E (*n* = 17, 7.9%)	
		F (*n* = 0, 0.0%)		F (*n* = 6, 2.8%)	
		G (*n* = 1, 2.6%)		G (*n* = 4, 1.9%)	
		H (*n* = 2, 5.3%)		H (*n* = 2, 0.9%)	
	SeP (*n* = 8, 21.1%)	I (*n* = 3, 7.9%)	SeP (*n* = 80, 37.4%)	I (*n* = 7, 3.3%)	
		J (*n* = 2, 5.3%)		J (*n* = 30, 14.0%)	
		K (*n* = 0, 0.0%)		K (*n* = 11, 5.1%)	
		L (*n* = 3, 7.9%		L (*n* = 32, 15.0%)	
**Individual serological response classification - CA-125**	SeR (*n* = 13, 35.1%)	A (*n* = 2, 5.4%)	SeR (*n* = 44, 21.8%)	A (*n* = 9, 4.5%)	0.008 *
		B (*n* = 11, 3.0%)		B (*n* = 35, 17.3%)	
	SeS (*n* = 21, 56.8%)	C (*n* = 18, 48.6%)	SeS (*n* = 97, 48.0%)	C (*n* = 57, 28.2%)	
		D (*n* = 0, 0.0%)		D (*n* = 10, 5.0%)	
		E (*n* = 1, 2.7%)		E (*n* = 13, 6.4%)	
		F (*n* = 0, 0.0%)		F (*n* = 8, 4.0%)	
		G (*n* = 0, 0.0%)		G (*n* = 4, 2.0%)	
		H (*n* = 2, 5.4%)		H (*n* = 5, 2.5%)	
	SeP (*n* = 3, 8.1%)	I (*n* = 1, 2.7%)	SeP (*n* = 61, 30.2%)	I (*n* = 15, 7.4%)	
		J (*n* = 1, 2.7%)		J (*n* = 11, 5.4%)	
		K (*n* = 1, 2.7%)		K (*n* = 17, 8.4%)	
		L (*n* = 0, 0.0%)		L (*n* = 18, 8.9%)	
**Combined serological response classification**	SeR (*n* = 3, 7.9%)		SeR (*n* = 3, 1.4%)		<0.001 *
	SeS (*n* = 23, 60.5%)		SeS (*n* = 66, 30.3%)		
	SeP (*n* = 12, 31.6%)		SeP (*n* = 149, 68.3%)		

* *p*-value <0.05. Abbreviations: Cyfra 21.1, Cytokeratin 19 fragment antigen; CEA, CarcinoEmbryonic Antigen: CA-125, Cancer Antigen-125; SeR, serological remission; SeS,

serological stable/unknown significance; SeP, serological progression. [a] Missing data: CA-125 (n = 1).[b] Missing data: CEA (n = 4), CA-125 (n = 16).

Table S8. Overview of the added value of the three main serological response patterns for the individual and combined serological response classifications in distinguishing patients with secondary treatment resistance who developed oligoprogression versus systemic progression.

	**Oligoprogression (*n* = 55) [a]**		**Systemic progression (*n* = 163) [b]**		***p*-value**
**Individual serological response classification - Cyfra 21.1**	SeR (n = 9, 16.4%)	A (n = 3, 5.5%)	SeR (n = 11, 6.7%)	A (n = 3, 1.8%)	<0.001 *
		B (n = 6, 10.9%)		B (n = 8, 4.9%)	
	SeS (n = 34, 61.8%)	C (n = 3, 5.5%)	SeS (n = 56, 34.4%)	C (n = 5, 3.1%)	
		D (n = 9, 16.4%)		D (n = 11, 6.7%)	
		E (n = 8, 14.5%)		E (n = 13, 8.0%)	
		F (n = 3, 5.5%)		F (n = 12, 7.4%)	
		G (n = 4, 7.3%)		G (n = 9, 5.5%)	
		H (n = 7, 12.7%)		H (n = 6, 3.7%)	
	SeP (n = 12, 21.8%)	I (n = 1, 1.8%)	SeP (n = 96, 58.9%)	I (n = 6, 3.7%)	
		J (n = 0, 0.0%)		J (n = 30, 18.4%)	
		K (n = 6, 10.9%)		K (n = 16, 9.8%)	
		L (n = 5, 9.1%)		L (n = 44, 27.0%)	
**Individual serological response classification - CEA**	SeR (n = 9, 16.7%)	A (n = 2, 3.6%)	SeR (n = 16, 10.0%)	A (n = 3, 1.8%)	0.09
		B (n = 7, 12.7%)		B (n = 13, 8.0%)	
	SeS (n = 31, 57.4%)	C (n = 17, 30.9%)	SeS (n = 78, 48.8%)	C (n = 49, 30.1%)	
		D (n = 4, 7.3%)		D (n = 10, 6.1%)	
		E (n = 6, 10.9%)		E (n = 11, 6.7%)	
		F (n = 1, 1.8%)		F (n = 5, 3.1%)	
		G (n = 2, 3.6%)		G (n = 2, 1.2%)	
		H (n = 1, 1.8%)		H (n = 1, 0.6%)	
	SeP (n = 14, 25.9%)	I (n = 1, 1.8%)	SeP (n = 66, 41.3%)	I (n = 6, 3.7%)	
		J (n = 5, 9.1%)		J (n = 25, 15.3%)	
		K (n = 2, 3.6%)		K (n = 9, 5.5%)	
		L (n = 6, 10.9%)		L (n = 26, 16.0%)	
**Individual serological response classification - CA-125**	SeR (n = 12, 23.5%)	A (n = 3, 5.5%)	SeR (n = 32, 21.2%)	A (n = 6, 3.7%)	0.02 *
		B (n = 9, 16.4%)		B (n = 26, 16.0%)	
	SeS (n = 31, 60.8%)	C (n = 22, 40.0%)	SeS (n = 66, 43.7%)	C (n = 35, 21.5%)	
		D (n = 3, 5.5%)		D (n = 7, 4.3%)	
		E (n = 4, 7.3%)		E (n = 9, 5.5%)	
		F (n = 0, 0.0%)		F (n = 8, 4.9%)	
		G (n = 0, 0.0%)		G (n = 4, 2.5%)	
		H (n = 2, 3.6%)		H (n = 3, 1.8%)	
	SeP (n = 8, 15.7%)	I (n = 2, 3.6%)	SeP (n = 53, 35.1%)	I (n = 13, 8.0%)	
		J (n = 2, 3.6%)		J (n = 9, 5.5%)	
		K (n = 3, 5.5%)		K (n = 14, 8.6%)	
		L (n = 1, 1.8%)		L (n = 17, 10.4%)	
**Combined serological response classification**	SeR (n = 1, 1.8%)		SeR (n = 2, 1.2%)		<0.001 *
	SeS (n = 29, 52.7%)		SeS (n = 37, 22.7%)		
	SeP (n = 25, 45.5%)		SeP (n = 124, 76.1%)		

* p-value < 0.05. Abbreviations: Cyfra 21.1, Cytokeratin 19 fragment antigen; CEA, CarcinoEmbryonic Antigen: CA-125, Cancer Antigen-125; SeR, serological remission; SeS, serological stable/unknown significance; SeP, serological progression. [a] Missing data: CEA (n = 1), CA-125 (n = 4). [b] Missing data: CEA (n = 3), CA-125 (n = 12).

Table S9. Overview of the added value of the three main serological response patterns for the individual and combined serological response classifications in distinguishing patients with a durable response who received treatment for two years versus those who received treatment less than two years.

	**Two years of treatment (n = 21)**		**Less than two years of treatment (n = 17) [a]**		***p*-value**
**Individual serological response classification - Cyfra 21.1**	SeR (n = 6, 28.6%)	A (n = 3, 14.3%)	SeR (n = 2, 11.8%)	A (n = 1, 5.9%)	0.48
		B (n = 3, 14.3%)		B (n = 1, 5.9%)	
	SeS (n = 14, 66.7%)	C (n = 2, 9.5%)	SeS (n = 13, 76.5%)	C (n = 2, 11.8%)	
		D (n = 2, 9.5%)		D (n = 0, 0.0%)	
		E (n = 0, 0.0%)		E (n = 1, 5.9%)	
		F (n = 1, 4.8%)		F (n = 0, 0.0%)	
		G (n = 7, 33.3%)		G (n = 2, 11.8%)	
		H (n = 2, 9.5%)		H (n = 8, 47.1%)	
	SeP (n = 1, 4.8%)	I (n = 1, 4.8%)	SeP (n = 2, 11.8%)	I (n = 1, 5.9%)	
		J (n = 0, 0.0%)		J (n = 1, 5.9%)	
		K (n = 0, 0.0%)		K (n = 0, 0.0%)	
		L (n = 0, 0.0%)		L (n = 0, 0.0%)	
**Individual serological response classification - CEA**	SeR (n = 7, 33.3%)	A (n = 1, 4.8%)	SeR (n = 5, 29.4%)	A (n = 2, 11.8%)	0.63
		B (n = 6, 28.6%)		B (n = 3, 17.6%)	
	SeS (n = 11, 52.4%)	C (n = 5, 23.8%)	SeS (n = 7, 41.2%)	C (n = 6, 35.3%)	
		D (n = 2, 9.5%)		D (n = 0, 0.0%)	
		E (n = 1, 4.8%)		E (n = 1, 5.9%)	
		F (n = 0, 0.0%)		F (n = 0, 0.0%)	
		G (n = 1, 4.8%)		G (n = 0, 0.0%)	
		H (n = 2, 9.5%)		H (n = 0, 0.0%)	
	SeP (n = 3, 14.3%)	I (n = 2, 9.5%)	SeP (n = 5, 29.4%)	I (n = 1, 5.9%)	
		J (n = 0, 0.0%)		J (n = 2, 11.8%)	
		K (n = 0, 0.0%)		K (n = 0, 0.0%)	
		L (n = 1, 4.8%)		L (n = 2, 11.8%)	
**Individual serological response classification - CA-125**	SeR (n = 7, 35.0%)	A (n = 2, 9.5%)	SeR (n = 6, 35.3%)	A (n = 0, 0.0%)	0.89
		B (n = 5, 23.8%)		B (n = 6, 35.3%)	
	SeS (n = 12, 60.0%)	C (n = 10, 47.6%)	SeS (n = 9, 52.9%)	C (n = 8, 47.1%)	
		D (n = 0, 0.0%)		D (n = 0, 0.0%)	
		E (n = 1, 4.8%)		E (n = 0, 0.0%)	
		F (n = 0, 0.0%)		F (n = 0, 0.0%)	
		G (n = 0, 0.0%)		G (n = 0, 0.0%)	
		H (n = 1, 4.8%)		H (n = 1, 5.9%)	
	SeP (n = 1, 5.0%)	I (n = 1, 4.8%)	SeP (n = 2, 11.8%)	I (n = 0, 0.0%)	
		J (n = 0, 0.0%)		J (n = 1, 5.9%)	
		K (n = 0, 0.0%)		K (n = 1, 5.9%)	
		L (n = 0, 0.0%)		L (n = 0, 0.0%)	
**Combined serological**	SeR (n = 2, 9.5%)		SeR (n = 1, 5.9%)		0.47
	SeS		SeS		

**response classification**	(*n* = 14, 66.7%)		(*n* = 9, 52.9%)		
	SeP (*n* = 5, 23.8%)		SeP (*n* = 7, 41.2%)		

Abbreviations: Cyfra 21.1, Cytokeratin 19 fragment antigen; CEA, CarcinoEmbryonic Antigen: CA-125, Cancer Antigen-125; SeR, serological remission; SeS, serological stable/unknown significance; SeP, serological progression. [a] Missing data: CA-125 (*n* = 1).

Table S10. Overview of the added value of the three main serological response patterns for the individual and combined serological response classifications in distinguishing patients with a durable response who progressed after two years versus those who did not.

	**Progression after two years (*n* = 13)** [a]		**No progression after two years (*n* = 25)**		***p*-value**
**Individual serological response classification - Cyfra 21.1**	SeR (*n* = 4, 30.8%)	A (*n* = 2, 15.4%)	SeR (*n* = 4, 16.0%)	A (*n* = 2, 8.0%)	0.19
		B (*n* = 2, 15.4%)		B (*n* = 2, 8.0%)	
	SeS (*n* = 7, 53.8%)	C (*n* = 1, 7.7%)	SeS (*n* = 20, 80.0%)	C (*n* = 3, 12.0%)	
		D (*n* = 0, 0.0%)		D (*n* = 2, 8.0%)	
		E (*n* = 0, 0.0%)		E (*n* = 1, 4.0%)	
		F (*n* = 0, 0.0%)		F (*n* = 1, 4.0%)	
		G (*n* = 3, 23.1%)		G (*n* = 6, 24.0%)	
		H (*n* = 3, 23.1%)		H (*n* = 7, 28.0%)	
	SeP (*n* = 2, 15.4%)	I (*n* = 1, 7.7%)	SeP (*n* = 1, 4.0%)	I (*n* = 1, 4.0%)	
		J (*n* = 1, 7.7%)		J (*n* = 0, 0.0%)	
		K (*n* = 0, 0.0%)		K (*n* = 0, 0.0%)	
		L (*n* = 0, 0.0%)		L (*n* = 0, 0.0%)	
**Individual serological response classification - CEA**	SeR (*n* = 4, 30.8%)	A (*n* = 0, 0.0%)	SeR (*n* = 8, 32.0%)	A (*n* = 3,12.0%)	0.61
		B (*n* = 4, 30.8%)		B (*n* = 5, 20.0%)	
	SeS (*n* = 5, 38.5%)	C (*n* = 3, 23.1%)	SeS (*n* = 13, 52.0%)	C (*n* = 8, 32.0%)	
		D (*n* = 0, 0.0%)		D (*n* = 2, 8.0%)	
		E (*n* = 0, 0.0%)		E (*n* = 2, 8.0%)	
		F (*n* = 0, 0.0%)		F (*n* = 0, 0.0%)	
		G (*n* = 1, 7.7%)		G (*n* = 0, 0.0%)	
		H (*n* = 1, 7.7%)		H (*n* = 1, 4.0%)	
	SeP (*n* = 4, 30.8%)	I (*n* = 1, 7.7%)	SeP (*n* = 4, 16.0%)	I (*n* = 2, 8.0%)	
		J (*n* = 2, 15.4%)		J (*n* = 0, 0.0%)	
		K (*n* = 0, 0.0%)		K (*n* = 0, 0.0%)	
		L (*n* = 1, 7.7%)		L (*n* = 2, 8.0%)	
**Individual serological response classification - CA-125**	SeR (*n* = 4, 33.3%)	A (*n* = 0, 0.0%)	SeR (*n* = 9, 36.0%)	A (*n* = 2, 8.0%)	0.50
		B (*n* = 4, 33.3%)		B (*n* = 7, 28.0%)	
	SeS (*n* = 6, 50.0%)	C (*n* = 5, 41.7%)	SeS (*n* = 15, 60.0%)	C (*n* = 13, 52.0%)	
		D (*n* = 0, 0.0%)		D (*n* = 0, 0.0%)	
		E (*n* = 0, 0.0%)		E (*n* = 1, 4.0%)	
		F (*n* = 0, 0.0%)		F (*n* = 0, 0.0%)	
		G (*n* = 0, 0.0%)		G (*n* = 0, 0.0%)	
		H (*n* = 1, 8.3%)		H (*n* = 1, 4.0%)	
	SeP (*n* = 2, 16.7%)	I (*n* = 1, 8.3%)	SeP (*n* = 1, 4.0%)	I (*n* = 0, 0.0%)	
		J (*n* = 1, 8.3%)		J (*n* = 0, 0.0%)	
		K (*n* = 0, 0.0%)		K (*n* = 1, 4.0%)	
		L (*n* = 0, 0.0%)		L (*n* = 0, 0.0%)	
**Combined serological**	SeR (*n* = 1, 7.7%)		SeR (*n* = 2, 8.0%)		0.39

**response classification**	SeS (n = 6, 46.2%)		SeS (n = 17, 68.0%)		
	SeP (n = 6, 46.2%)		SeP (n = 6, 24.0%)		

Abbreviations: Cyfra 21.1, Cytokeratin 19 fragment antigen; CEA, CarcinoEmbryonic Antigen: CA-125, Cancer Antigen-125; SeR, serological remission; SeS, serological stable/unknown significance; SeP, serological progression. [a] Missing data: CA-125 (n = 1).

Table S11. Overview of the added value of the three main serological response patterns for the individual and combined serological response classifications in distinguishing patients in whom treatment was discontinued due to irAEs versus those in whom this was not.

	**Treatment discontinuation due to irAEs (n = 41) [a]**		**No treatment discontinuation due to irAEs (n = 215) [b]**		***p*-value**
**Individual serological response classification - Cyfra 21.1**	SeR (n = 1, 2.4%)	A (n = 0, 0.0%)	SeR (n = 27, 12.6%)	A (n = 10, 4.7%)	0.10
		B (n = 1, 2.4%)		B (n = 17, 7.9%)	
	SeS (n = 23, 56.1%)	C (n = 2, 4.9%)	SeS (n = 94, 43.7%)	C (n = 10, 4.7%)	
		D (n = 4, 9.8%)		D (n = 18, 8.4%)	
		E (n = 3, 7.3%)		E (n = 19, 8.8%)	
		F (n = 3, 7.3%)		F (n = 13, 6.0%)	
		G (n = 4, 9.8%)		G (n = 18, 8.4%)	
		H (n = 7, 17.1%)		H (n = 16, 7.4%)	
	SeP (n = 17, 41.5%)	I (n = 3, 7.3%)	SeP (n = 94, 43.7%)	I (n = 6, 2.8%)	
		J (n = 2, 4.9%)		J (n = 29, 13.5%)	
		K (n = 3, 7.3%)		K (n = 19, 8.8%)	
		L (n = 9, 22.0%)		L (n = 40, 18.6%)	
**Individual serological response classification - CEA**	SeR (n = 5, 12.5%)	A (n = 2, 4.9%)	SeR (n = 32, 15.1%)	A (n = 6, 2.8%)	0.24
		B (n = 3, 7.3%)		B (n = 26, 12.1%)	
	SeS (n = 25, 62.5%)	C (n = 16, 39.0%)	SeS (n = 102, 48.1%)	C (n = 61, 28.4%)	
		D (n = 2, 4.9%)		D (n = 14, 6.5%)	
		E (n = 4, 9.8%)		E (n = 15, 7.0%)	
		F (n = 2, 4.9%)		F (n = 4, 1.9%)	
		G (n = 2, 4.9%)		G (n = 5, 2.3%)	
		H (n = 0, 0.0%)		H (n = 3, 1.4%)	
	SeP (n = 10, 25.0%)	I (n = 1, 2.4%)	SeP (n = 78, 36.8%)	I (n = 10, 4.7%)	
		J (n = 0, 0.0%)		J (n = 31, 14.4%)	
		K (n = 1, 2.4%)		K (n = 8, 3.7%)	
		L (n = 3, 7.3%)		L (n = 29, 13.5%)	
**Individual serological response classification - CA-125**	SeR (n = 11, 27.5%)	A (n = 1, 2.4%)	SeR (n = 46, 23.1%)	A (n = 10, 4.7%)	0.86
		B (n = 10, 24.4%)		B (n = 36, 16.7%)	
	SeS (n = 19, 47.5%)	C (n = 9, 22.0%)	SeS (n = 99, 49.7%)	C (n = 66, 30.7%)	
		D (n = 2, 4.9%)		D (n = 8, 3.7%)	
		E (n = 1, 2.4%)		E (n = 13, 6.0%)	
		F (n = 2, 4.9%)		F (n = 6, 2.8%)	
		G (n = 3, 7.3%)		G (n = 1, 0.5%)	
		H (n = 2, 4.9%)		H (n = 5, 2.3%)	
	SeP (n = 10, 25.0%)	I (n = 1, 2.4%)	SeP (n = 54, 27.1%)	I (n = 15, 7.0%)	
		J (n = 1, 2.4%)		J (n = 11, 5.1%)	
		K (n = 5, 12.2%)		K (n = 13, 6.0%)	
		L (n = 3, 7.3%)		L (n = 15, 7.0%)	
	SeR		SeR		>0.99

**Combined serological response classification**	(n = 1, 2.4%)	(n = 5, 2.3%)
	SeS (n = 14, 34.1%)	SeS (n = 75, 34.9%)
	SeP (n = 26, 63.4%)	SeP (n = 135, 62.8%)

Abbreviations: irAEs, immune-related adverse events; Cyfra 21.1, Cytokeratin 19 fragment antigen; CEA, CarcinoEmbryonic Antigen: CA-125, Cancer Antigen-125; SeR, serological remission; SeS, serological stable/unknown significance; SeP, serological progression. [a] Missing data: CEA (n = 1), CA-125 (n = 1). [b] Missing data: CEA (n = 3), CA-125 (n = 16).

Table S12. Overview of the median progression-free survival and overall survival associated with the three serological response patterns for the individual and combined serological response classifications.

	**Median PFS in months (95% CI)**	**Median OS in months (95% CI)**
**Cyfra 21.1**		
SeR	9.0 (1.5–16.6)	35.0 (28.9–41.1)
SeS	9.0 (7.0–10.9)	28.2(14.8–41.6)
SeP	6.4 (5.7–7.1)	14.5 (12.4–16.6)
**CEA**		
SeR	10.8 (7.2–14.4)	91.1 (5.6–176.6)
SeS	7.6 (6.5–8.6)	19.6 (16.1–23.2)
SeP	6.9 (5.8–7.9)	17.7 (14.8–20.6)
**CA-125**		
SeR	9.0 (7.8–10.1)	25.5 (16.2–34.7)
SeS	7.2 (6.5–8.0)	21.8 (17.4–26.2)
SeP	6.8 (5.5–8.2)	13.6 (11.0–16.1)
**Combined serological response classification**		
SeR	10.8 (0.0–43.7)	NR (NR-NR)
SeS	8.3 (6.5–10.1)	29.7 (15.5–43.9)
SeP	6.9 (6.1–7.8)	16.5 (14.0–18.9)

Abbreviations: PFS, progression-free survival; CI, confidence interval; OS, overall survival; Cyfra 21.1, Cytokeratin 19 fragment antigen; CEA, CarcinoEmbryonic Antigen: CA-125, Cancer Antigen-125; NR, not reached; SeR, serological remission; SeS, serological stable/unknown significance; SeP, serological progression.

Table S13. Overview of the results obtained with the log-rank test when comparing the progression-free survival between the three serological response patterns for the individual and combined serological response classifications.

	**SeR**		**SeS**		**SeP**	
	χ^2	***p*-value**	χ^2	***p*-value**	χ^2	***p*-value**
**Cyfra 21.1**						
SeR			0.132	0.72	10.373	0.001 *
SeS	0.132	0.72			29.847	<0.001 *
SeP	10.373	0.001 *	29.847	<0.001 *		
**CEA**						
SeR			4.251	0.04 *	8.388	0.004 *
SeS	4.251	0.04 *			1.602	0.21
SeP	8.388	0.004 *	1.602	0.21		

**CA-125**						
SeR			0.844	0.36	7.939	0.005 *
SeS	0.844	0.36			3.889	0.05 *
eP	7.939	0.005 *	3.889	0.05 *		
**Combined serological response classification**						
SeR			0.493	0.48	4.015	0.05 *
SeS	0.493	0.48			14.999	<0.001 *
SeP	4.015	0.05 *	14.999	<0.001 *		

* *p*-value < 0.05. Abbreviations: Cyfra 21.1, Cytokeratin 19 fragment antigen; CEA, CarcinoEmbryonic Antigen: CA-125, Cancer Antigen-125; SeR, serological remission; SeS, serological stable/unknown significance; SeP, serological progression.

Table S14. Overview of the results obtained with the log-rank test when comparing the survival between the three serological response patterns for the individual and combined serological response classifications.

	**SeR**		**SeS**		**SeP**	
	χ^2	***p*-value**	χ^2	***p*-value**	χ^2	***p*-value**
**Cyfra 21.1**						
SeR			0.321	0.57	17.378	<0.001 *
SeS	0.321	0.57			35.073	<0.001 *
SeP	17.378	<0.001 *	35.073	<0.001 *		
**CEA**						
SeR			10.109	0.001 *	13.899	<0.001 *
SeS	10.109	0.001 *			1.255	0.26
SeP	13.899	<0.001 *	1.255	0.26		
**CA-125**						
SeR			0.905	0.34	16.814	<0.001 *
SeS	0.905	0.34			13.672	<0.001 *
SeP	16.814	<0.001 *	13.672	<0.001 *		
**Combined serological response classification**						
SeR			1.909	0.17	5.806	0.02 *
SeS	1.909	0.17			17.339	<0.001 *
SeP	5.806	0.02 *	17.339	<0.001 *		

* *p*-value < 0.05. Abbreviations: Cyfra 21.1, Cytokeratin 19 fragment antigen; CEA, CarcinoEmbryonic Antigen: CA-125, Cancer Antigen-125; SeR, serological remission; SeS, serological stable/unknown significance; SeP, serological progression.

Table S15. Overview of the median progression-free survival and overall survival associated with the three main response patterns for the STM dynamics subclassified as SeS.

	**Median PFS in months (95% CI)**	**Median OS in months (95% CI)**
**Cyfra 21.1**		
C	7.6 (6.1–9.0)	21.9 (10.1–33.6)
D	6.8 (6.5–7.2)	18.5 (15.6–21.4)
E	5.4 (5.1–5.8)	15.5 (9.2–21.8)
F	9.0 (4.5–13.5)	15.4 (14.6–16.2)
G	21.8 (14.6–29.0)	54.8 (41.4–68.2)
H	21.9 (18.4–25.3)	71.9 (41.8–101.9)

**CEA**		
C	8.1 (6.9–9.3)	18.6 (14.0–23.3)
D	7.4 (6.6–8.3)	19.9 (17.9–22.0)
E	6.0 (5.3–6.7)	21.0 (12.8–29.2)
F	6.8 (2.3–11.3)	11.5 (4.3–18.6)
G	9.0 (7.9–10.2)	25.5 (3.2–47.8)
H	6.9 (0.0–35.0)	NR (NR-NR)
**CA-125**		
C	8.1 (6.9–9.3)	33.0 (15.7–50.4)
D	4.8 (3.5–6.1)	10.9 (6.4–15.5)
E	5.4 (4.9–5.9)	11.8 (9.2–14.4)
F	6.1 (4.7–7.6)	15.4 (9.6–21.2)
G	4.2 (0.0–9.8)	7.8 (0.0–21.4)
H	21.2 (5.6–36.7)	64.2 (0.0–134.8)

Abbreviations: STM, serum tumor marker; SeS, serological stable/unknown significance; PFS, progression-free survival; CI, confidence interval; OS, overall survival; Cyfra 21.1, Cytokeratin 19 fragment antigen; CEA, CarcinoEmbryonic Antigen: CA-125, Cancer Antigen-125; NR, not reached.

Table S16. Overview of the results obtained with the log-rank test when comparing the progression-free survival between the STM dynamics subclassified as SeS.

	**C**		**D**		**E**		**F**		**G**		**H**	
	χ^2	***p*-value**	χ^2	***p*-value**	χ^2	***p*-value**	χ^2	***p*-value**	χ^2	***p*-value**	χ^2	***p*-value**
**Cyfra 21.1**												
C			2.097	0.15	5.673	0.02 *	0.768	0.38	0.440	0.51	1.296	0.26
D	2.097	0.15			2.452	0.117	0.365	0.55	8.310	0.004 *	9.651	0.002 *
E	5.673	0.02 *	2.452	0.12			4.521	0.03 *	18.978	<0.001 *	20.225	<0.001 *
F	0.768	0.38	0.365	0.55	4.521	0.03 *			7.528	0.006 *	9.461	0.002 *
G	0.440	0.51	8.310	0.004 *	18.978	<0.001 *	7.528	0.006 *			0.337	0.56
H	1.296	0.26	9.651	0.002 *	20.225	<0.001 *	9.461	0.002 *	0.337	0.56		
**CEA**												
C			0.010	0.92	3.338	0.07	1.327	0.25	0.001	0.98	0.871	0.35
D	0.010	0.92			1.823	0.18	1.091	0.30	0.003	0.96	0.493	0.48
E	3.338	0.07	1.823	0.18			0.302	0.58	0.892	0.35	1.872	0.17
F	1.327	0.25	1.091	0.30	0.302	0.58			0.420	0.52	1.741	0.19
G	0.001	0.98	0.003	0.96	0.892	0.35	0.420	0.52			0.967	0.33
H	0.871	0.35	0.493	0.48	1.872	0.17	1.741	0.19	0.967	0.33		
**CA-125**												
C			16.873	<0.001 *	8.641	0.003 *	3.503	0.06	1.351	0.25	0.173	0.68
D	16.873	<0.001 *			1.178	0.28	2.605	0.11	1.467	0.23	8.769	0.003 *
E	8.641	0.003 *	1.178	0.28			0.388	0.53	0.059	0.81	3.809	0.05
F	3.503	0.06	2.605	0.11	0.388	0.53			0.224	0.64	4.906	0.03 *
G	1.351	0.25	1.467	0.23	0.059	0.81	0.224	0.64			3.749	0.05
H	0.173	0.68	8.769	0.003 *	3.809	0.05	4.906	0.03 *	3.749	0.05		

* *p*-value < 0.05. Abbreviations: STM, serum tumor marker; SeS, serological stable/unknown significance; PFS, progression-free survival; CI, confidence interval; OS, overall survival; Cyfra 21.1, Cytokeratin 19 fragment antigen; CEA, CarcinoEmbryonic Antigen: CA-125, Cancer Antigen-125.

Table S17. Overview of the results obtained with the log-rank test when comparing the overall survival between the STM dynamics subclassified as SeS.

	C		D		E		F		G		H	
	χ^2	*p*-value	χ^2	*p*-value	χ^2	*p*-value	χ^2	*p*-value	χ^2	*p*-value	χ^2	*p*-value
**Cyfra 21.1**												
C			0.035	0.85	1.221	0.27	1.247	0.26	2.690	0.10	7.002	0.008 *
D	0.035	0.85			1.172	0.28	1.329	0.25	2.245	0.13	8.023	0.005 *
E	1.221	0.27	1.172	0.28			0.012	0.92	6.970	0.008 *	18.141	<0.001 *
F	1.247	0.26	1.329	0.25	0.012	0.92			7.213	0.007 *	14.155	<0.001 *
G	2.690	0.10	2.245	0.13	6.970	0.008 *	7.213	0.007 *			1.739	0.19
H	7.002	0.008 *	8.023	0.005 *	18.141	<0.00 1*	14.155	<0.001 *	1.739	0.19		
**CEA**												
C			0.000	0.99	0.726	0.39	0.966	0.33	0.156	0.69	2.771	0.10
D	0.000	0.99			0.348	0.56	0.969	0.33	0.132	0.72	2.265	0.13
E	0.726	0.39	0.348	0.56			0.456	0.50	0.727	0.39	4.220	0.04 *
F	0.966	0.33	0.969	0.33	0.456	0.50			0.869	0.35	2.761	0.10
G	0.156	0.69	0.132	0.72	0.727	0.39	0.869	0.35			1.423	0.23
H	2.771	0.10	2.265	0.13	4.220	0.04 *	2.761	0.10	1.423	0.23		
**CA-125**												
C			24.902	<0.001 *	15.112	<0.001 *	9.763	0.002 *	6.042	0.01 *	0.565	0.45
D	24.902	<0.001 *			0.744	0.39	1.117	0.29	0.423	0.52	7.160	0.007 *
E	15.112	<0.001 *	0.744	0.39			0.114	0.74	0.002	0.96	5.257	0.02 *
F	9.763	0.002 *	1.117	0.29	0.114	0.74			0.171	0.68	4.507	0.03 *
G	6.042	0.01 *	0.423	0.52	0.002	0.96	0.171	0.68			3.259	0.07
H	0.565	0.45	7.160	0.007 *	5.257	0.02 *	4.507	0.03 *	3.259	0.07		

* *p*-value < 0.05. Abbreviations: STM, serum tumor marker; SeS, serological stable/unknown significance; PFS, progression-free survival; CI, confidence interval; OS, overall survival; Cyfra 21.1, Cytokeratin 19 fragment antigen; CEA, CarcinoEmbryonic Antigen: CA-125, Cancer Antigen-125.

Table S18. Overview of the results obtained with the multivariable Cox regression analysis when including the individual serological response classification for Cyfra 21.1.

Variable [a]	Patients, *n* = 256 (100.0%)			
	PFS		OS	
	HR (95% CI)	*p*-value	HR (95% CI)	*p*-value
Individual serological response classification for Cyfra 21.1	1.982 (1.356–2.897)	<0.001 *	2.654 (1.823–3.863)	<0.001 *
**General characteristics**				
Age	0.996 (0.968–1.024)	0.76	0.980 (0.950–1.011)	0.21
Gender	0.628 (0.395–0.998)	0.05 *	0.704 (0.424–1.168)	0.17
BMI	1.110 (1.047–1.177)	<0.001 *	1.049 (0.984–1.118)	0.14
ECOG PS	1.000 (0.437–2.287)	>0.99	2.154 (0.890–5.212)	0.09
Ethnicity	0.977 (0.395–2.417)	0.96	0.945 (0.298–1.118)	0.92
Haemoglobin level	1.986 (0.619–6.369)	0.25	0.949 (0.281–3.211)	0.93
Albumin level	1.431 (0.838–2.444)	0.19	2.252 (1.234–4.079)	0.007 *
Kidney function	1.153 (0.560–2.374)	0.70	1.330 0.576–3.070)	0.50
**Smoking**				
Smoking status	0.696 (0.412–1.176)	0.18	1.023 (0.584–1.793)	0.94
Pack years	0.985 (0.972–0.999)	0.04 *	0.987 (0.972–1.002)	0.08
**Comorbidities**				
COPD	1.353 (0.809–2.263)	0.25	0.804 (0.453–1.425)	0.46
Diabetes mellitus	0.661 (0.332–1.313)	0.24	0.831 (0.380–1.814)	0.64

Auto-immune disease	0.962 (0.409–2.261)	0.93	1.630 (0.641–4.144)	0.31
Kidney disease	2.870 (1.086–7.583)	0.03 *	0.813 (0.249–2.652)	0.73
Liver disease	10.828 (0.871–134.657)	0.06	5.849 (0.452–75.738)	0.18
Cancer	1.319 (0.351–4.958)	0.68	3.633 (0.894–17.763)	0.07
**Medication use**				
Immunosuppressants	2.526 (0.947–6.738)	0.06	1.314 (0.478–3.608)	0.60
Aspirin/NSAIDs	1.016 (0.552–1.870)	0.96	1.185 (0.643–2.182)	0.59
Anticoagulants	0.879 (0.451–1.714)	0.71	1.199 (0.597–2.411)	0.61
Proton pump inhibitors	1.120 (0.669–1.874)	0.67	1.628 (0.904–2.932)	0.11
Antibiotics < 3 months	0.248 (0.100–0.615)	0.003 *	0.496 (0.191–1.291)	0.15
**Tumor characteristics**				
Histology	0.980 (0.903–1.063)	0.63	0.973 (0.885–1.071)	0.58
PD-L1 expression	1.288 (0.966–1.717)	0.09	0.910 (0.661–1.253)	0.56
Mutation status				
KRAS positive	0.444 (0.262–0.753)	0.003 *	0.736 (0.416–1.305)	0.30
EGFR positive	0.497 (0.242–1.021)	0.06	0.398 (0.165–0.963)	0.04 *
BRAF positive	0.633 (0.253–1.586)	0.33	0.635 (0.205–1.967)	0.43
ALK positive	1.682 (0.155–18.238)	0.67	4.127 (0.343–49.676)	0.26
Lung cancer stage	2.644 (1.127–6.208)	0.03 *	0.958 (0.348–2.642)	0.94
Localization of distant metastases				
Brain	1.465 (0.698–3.073)	0.31	1.451 (0.663–3.178)	0.35
Bone	1.308 (0.757–2.261)	0.34	1.626 (0.889–2.976)	0.12
Liver	0.795 (0.369–1.711)	0.56	0.782 (0.331–1.847)	0.58
Adrenal gland(s)	0.387 (0.218–0.686)	0.001 *	0.557 (0.292–1.061)	0.08
**Treatment**				
Current	2.350 (1.328–4.157)	0.003 *	1.551 (0.805–2.990)	0.19
Line of treatment	0.962 (0.520–1.778)	0.90	1.401 (0.705–2.786)	0.34
**Site of inclusion**	3.473 (1.475–8.179)	0.004 *	1.157 (0.474–2.820)	0.75

* *p*-value < 0.05. [a] Missing data: BMI (*n* = 1), ethnicity (*n* = 8), albumin level (*n* = 10), kidney function (*n* = 4), smoking status (*n* = 12), pack years (*n* = 46), histology (*n* = 4), PD-L1 expression (*n* = 43), KRAS status (*n* = 25), EGFR status (*n* = 23), BRAF status (*n* = 13), ALK status (*n* = 28), lung cancer stage (*n* = 5), brain metastases (*n* = 5), bone metastases (*n* = 5), liver metastases (*n* = 5), adrenal gland(s) metastases (*n* = 5), baseline Cyfra 21.1 levels (*n* = 59), baseline CEA levels (*n* = 60), baseline CA-125 levels (*n* = 66). Abbreviations: Cyfra 21.1, Cytokeratin 19 fragment antigen; PFS, progression-free survival; OS, overall survival; HR, hazard ratio; CI, confidence interval; BMI, body mass index; ECOG PS, Eastern Cooperative Oncology Group Performance Score; COPD, Chronic Obstructive Pulmonary Disease; NSAIDs, non-steroidal anti-inflammatory drugs; PD-L1, programmed death-ligand 1; KRAS, Kirsten rat sarcoma virus; EGFR, epidermal growth factor receptor; BRAF, B-Raf Proto-oncogene; ALK, anaplastic lymphoma kinase.

Table S19. Overview of the results obtained with the multivariable Cox regression analysis when including the individual serological response classification for CEA.

Variable [a]	Patients, *n* = 252 (98.4%)			
	PFS		OS	
	HR (95% CI)	*p*-value	HR (95% CI)	*p*-value
Individual serological response classification for CEA	1.191 (0.885–1.602)	0.25	1.826 (1.290–2.583)	<0.001 *
**General characteristics**				

Age	0.993 (0.966–1.021)	0.61	0.990 (0.960–1.021)	0.52
Gender	0.612 (0.389–0.963)	0.03 *	0.730 (0.453–1.176)	0.20
BMI	1.117 (1.054–1.183)	<0.001 *	1.048 (0.985–1.114)	0.14
ECOG PS	0.880 (0.380–2.041)	0.77	2.291 (0.943–5.565)	0.07
Ethnicity	1.014 (0.404–2.545)	0.98	1.130 (0.359–3.554)	0.83
Haemoglobin level	1.651 (0.525–5.193)	0.39	1.003 (0.301–3.345)	0.10
Albumin level	1.594 (0.919–2.766)	0.10	2.278 (1.237–4.194)	0.008 *
Kidney function	1.350 (0.658–2.772)	0.41	1.640 (0.716–3.758)	0.24
**Smoking**				
Smoking status	0.631 (0.384–1.039)	0.07	0.968 (0.587–1.596)	0.90
Pack years	0.987 (0.974–1.001)	0.06	0.988 (0.973–1.002)	0.10
**Comorbidities**				
COPD	1.284 (0.764–2.158)	0.35	0.690 (0.383–1.243)	0.22
Diabetes mellitus	0.631 (0.324–1.231)	0.18	0.696 (0.327–1.480)	0.35
Auto-immune disease	0.970 (0.408–2.307)	0.94	1.684 (0.664–4.270)	0.27
Kidney disease	2.764 (1.048–7.291)	0.04 *	0.906 (0.289–2.835)	0.87
Liver disease	16.630 (1.269–217.856)	0.03 *	8.539 (0.618–118.010)	0.11
Cancer	0.936 (0.247–3.547)	0.92	1.948 (0.485–7.833)	0.35
**Medication use**				
Immunosuppressants	3.426 (1.318–8.905)	0.01 *	1.788 (0.681–4.698)	0.24
Aspirin/NSAIDs	1.154 (0.630–2.116)	0.64	1.378 (0.751–2.529)	0.30
Anticoagulants	1.156 (0.596–2.243)	0.67	1.663 (0.839–3.294)	0.15
Proton pump inhibitors	0.888 (0.531–1.486)	0.65	1.124 (0.628–2.009)	0.69
Antibiotics < 3 months	0.224 (0.091–0.551)	0.001 *	0.325 (0.126–0.834)	0.02 *
**Tumor characteristics**				
Histology	0.968 (0.893–1.050)	0.44	0.975 (0.889–1.069)	0.59
PD-L1 expression	1.239 (0.919–1.670)	0.16	1.079 (0.771–1.511)	0.66
Mutation status				
KRAS positive	0.497 (0.295–0.837)	0.009 *	0.764 (0.426–1.368)	0.37
EGFR positive	0.507 (0.247–1.040)	0.06	0.434 (0.183–1.029)	0.06
BRAF positive	0.457 (0.190–1.103)	0.08	0.412 (0.142–1.196)	0.10
ALK positive	2.323 (0.206–26.244)	0.50	2.944 (0.237–36.626)	0.40
Lung cancer stage	2.705 (1.146–6.381)	0.02*	1.102 (0.402–3.025)	0.85
Localization of distant metastases				
Brain	1.235 (0.595–2.563)	0.57	1.557 (0.728–3.333)	0.25
Bone	1.442 (0.829–2.505)	0.20	1.389 (0.755–2.557)	0.29
Liver	0.859 (0.408–1.811)	0.69	1.013 (0.453–2.265)	0.98
Adrenal gland(s)	0.396 (0.220–0.713)	0.002 *	0.587 (0.312–1.105)	0.10
**Treatment**				
Current	2.943 (1.616–5.360)	<0.001 *	2.785 (1.395–5.561)	0.004 *
Line of treatment	0.922 (0.506–1.681)	0.79	1.347 (0.698–2.598)	0.38
**Site of inclusion**	0.291 (0.121–0.701)	0.006 *	0.989 (0.403–2.429)	0.98

* p-value < 0.05. [a] Missing data: BMI (n = 1), ethnicity (n = 8), albumin level (n = 10), kidney function (n = 4), smoking status (n = 12), pack years (n = 46), histology (n = 4), PD-L1 expression (n = 43), KRAS status (n = 25), EGFR status (n = 23), BRAF status (n = 13), ALK status (n = 28), lung cancer stage (n = 5), brain metastases (n = 5), bone metastases (n = 5), liver metastases (n = 5), adrenal gland(s) metastases (n = 5), baseline Cyfra 21.1 levels (n = 59), baseline CEA levels (n = 60), baseline CA-125 levels (n = 66). Abbreviations: CEA, CarcinoEmbryonic Antigen; PFS, progression-free survival; OS, overall survival; HR, hazard ratio; CI, confidence interval; BMI, body mass index; ECOG PS, Eastern

Cooperative Oncology Group Performance Score; COPD, Chronic Obstructive Pulmonary Disease; NSAIDs, non-steroidal anti-inflammatory drugs; PD-L1, programmed death-ligand 1; KRAS, Kirsten rat sarcoma virus; EGFR, epidermal growth factor receptor; BRAF, B-Raf Proto-oncogene; ALK, anaplastic lymphoma kinase.

Table S20. Overview of the results obtained with the multivariable Cox regression analysis when including the individual serological response classification for CA-125.

Variable [a]	Patients, *n* = 239 (93.4%)			
	PFS		OS	
	HR (95% CI)	*p*-value	HR (95% CI)	*p*-value
Individual serological response classification for CA-125	1.415 (0.999–2.004)	0.051	1.774 (1.213–2.595)	0.003 *
**General characteristics**				
Age	1.005 (0.976–1.036)	0.73	0.995 (0.962–1.028)	0.76
Gender	0.681 (0.423–1.096)	0.11	0.860 (0.509–1.454)	0.57
BMI	1.117 (1.046–1.194)	0.001 *	1.017 (0.949–1.090)	0.62
ECOG PS	0.831 (0.354–1.951)	0.67	1.302 (0.512–3.309)	0.58
Ethnicity	0.783 (0.296–2.069)	0.62	0.795 (0.236–2.679)	0.71
Haemoglobin level	1.037 (0.272–3.957)	0.96	0.674 (0.181–2.510)	0.56
Albumin level	1.176 (0.635–2.178)	0.61	1.547 (0.777–3.077)	0.21
Kidney function	1.007 (0.484–2.095)	0.99	1.217 (0.526–2.815)	0.65
**Smoking**				
Smoking status	0.499 (0.286–0.871)	0.01 *	0.626 (0.351–1.118)	0.11
Pack years	0.994 (0.977–1.011)	0.46	0.999 (0.982–1.017)	0.95
**Comorbidities**				
COPD	1.586 (0.906–2.779)	0.11	1.176 (0.645–2.145)	0.60
Diabetes mellitus	0.549 (0.251–1.201)	0.13	0.845 (0.370–1.928)	0.69
Auto-immune disease	1.361 (0.537–3.451)	0.52	2.010 (0.780–5.177)	0.15
Kidney disease	2.720 (1.022–7.241)	0.05 *	0.865 (0.288–2.600)	0.80
Liver disease	8.151 (0.621–106.903)	0.11	6.064 (0.446–82.409)	0.18
Cancer	1.260 (0.252–6.299)	0.78	2.585 (0.496–13.482)	0.26
**Medication use**				
Immunosuppressants	3.736 (1.445–9.659)	0.007 *	2.287 (0.857–6.106)	0.10
Aspirin/NSAIDs	1.078 (0.549–2.116)	0.83	1.066 (0.544–2.086)	0.85
Anticoagulants	1.547 (0.688–3.479)	0.29	2.190 (0.953–5.033)	0.07
Proton pump inhibitors	1.152 (0.646–2.055)	0.63	1.665 (0.868–3.194)	0.13
Antibiotics < 3 months	0.192 (0.070–0.528)	0.001 *	0.272 (0.097–0.763)	0.01 *
**Tumor characteristics**				
Histology	0.979 (0.900–1.065)	0.62	1.010 (0.922–1.107)	0.83
PD-L1 expression	1.046 (0.760–1.439)	0.78	0.804 (0.558–1.158)	0.24
Mutation status				
KRAS positive	0.522 (0.302–0.903)	0.02 *	0.772 (0.425–1.401)	0.40
EGFR positive	0.592 (0.278–1.260)	0.17	0.553 (0.226–1.353)	0.19
BRAF positive	0.455 (0.165–1.259)	0.13	0.498 (0.151–1.641)	0.25
ALK [b] positive	NA (NA-NA)	NA	NA (NA-NA)	NA
Lung cancer stage	3.766 (1.453–9.762)	0.006 *	2.519 (0.828–7.665)	0.10
Localization of distant metastases				
Brain	1.457 (0.678–3.131)	0.34	1.566 (0.694–3.533)	0.28

Bone	1.323 (0.746–2.345)	0.34	1.377 (0.724–2.617)	0.33
Liver	0.843 (0.375–1.897)	0.68	0.761 (0.303–1.916)	0.56
Adrenal gland(s)	0.395 (0.211–0.739)	0.004 *	0.640 (0.325–1.259)	0.20
**Treatment**				
Current	2.744 (1.494–5.039)	0.001 *	1.865 (0.919–3.786)	0.09
Line of treatment	1.338 (0.695–2.578)	0.38	1.811 (0.902–3.637)	0.10
**Site of inclusion**	0.352 (0.142–0.868)	0.02 *	1.265 (0.500–3.199)	0.62

* p-value < 0.05. [a] Missing data: BMI (n = 1), ethnicity (n = 8), albumin level (n = 10), kidney function (n = 4), smoking status (n = 12), pack years (n = 46), histology (n = 4), PD-L1 expression (n = 43), KRAS status (n = 25), EGFR status (n = 23), BRAF status (n = 13), ALK status (n = 28), lung cancer stage (n = 5), brain metastases (n = 5), bone metastases (n = 5), liver metastases (n = 5), adrenal gland(s) metastases (n = 5), baseline Cyfra 21.1 levels (n = 59), baseline CEA levels (n = 60), baseline CA-125 levels (n = 66). [b] Constant or Linearly Dependent Covariates: ALK = 0. Abbreviations: CA-125, Cancer Antigen-125; PFS, progression-free survival; OS, overall survival; HR, hazard ratio; CI, confidence interval; BMI, body mass index; ECOG PS, Eastern Cooperative Oncology Group Performance Score; COPD, Chronic Obstructive Pulmonary Disease; NSAIDs, non-steroidal anti-inflammatory drugs; PD-L1, programmed death-ligand 1; KRAS, Kirsten rat sarcoma virus; EGFR, epidermal growth factor receptor; BRAF, B-Raf Proto-oncogene; ALK, anaplastic lymphoma kinase; NA, not applicable.

Table S21. Overview of the results obtained with the multivariable Cox regression analysis when including the combined serological response classification.

Variable [a]	Patients, n = 256 (100.0%)			
	**PFS**		**OS**	
	**HR (95% CI)**	***p*-value**	**HR (95% CI)**	***p*-value**
Combined serological response classification	1.563 (1.039–2.353)	0.03 *	2.726 (1.682–4.418)	<0.001 *
**General characteristics**				
Age	0.992 (0.966–1.019)	0.57	0.982 (0.953–1.012)	0.23
Gender	0.652 (0.409–1.040)	0.07	0.762 (0.462–1.256)	0.29
BMI	1.106 (1.045–1.172)	<0.001 *	1.045 (0.983–1.111)	0.16
ECOG PS	0.894 (0.395–2.022)	0.79	1.960 (0.816–4.707)	0.13
Ethnicity	1.044 (0.414–2.632)	0.93	1.179 (0.366–3.795)	0.78
Haemoglobin level	1.880 (0.598–5.909)	0.28	1.072 (0.327–3.513)	0.91
Albumin level	1.655 (0.954–2.869)	0.07	3.230 (1.712–6.095)	<0.001 *
Kidney function	1.256 (0.613–2.573)	0.53	1.754 (0.782–3.934)	0.17
**Smoking**				
Smoking status	0.622 (0.380–1.019)	0.06	0.907 (0.536–1.532)	0.71
Pack years	0.988 (0.974–1.001)	0.08	0.991 (0.976–1.006)	0.24
**Comorbidities**				
COPD	1.305 (0.775–2.199)	0.32	0.819 (0.461–1.456)	0.50
Diabetes mellitus	0.645 (0.333–1.247)	0.19	0.752 (0.354–1.597)	0.46
Auto-immune disease	0.950 (0.407–2.216)	0.91	1.459 (0.607–3.511)	0.40
Kidney disease	2.721 (1.028–7.207)	0.04 *	1.083 (0.362–3.242)	0.89
Liver disease	11.199 (0.895–140.138)	0.06	6.870 (0.543–86.907)	0.14
Cancer	0.971 (0.259–3.635)	0.97	2.256 (0.564–9.017)	0.25
**Medication use**				
Immunosuppressants	2.826 (1.110–7.196)	0.03 *	1.521 (0.590–3.924)	0.39
Aspirin/NSAIDs	1.303 (0.719–2.360)	0.38	1.662 (0.909–3.037)	0.10
Anticoagulants	1.161 (0.604–2.229)	0.65	1.631 (0.837–3.179)	0.15

Proton pump inhibitors	0.899 (0.541–1.492)	0.68	1.029 (0.577–1.836)	0.92
Antibiotics < 3 months	0.219 (0.088–0.541)	0.001 *	0.339 (0.132–0.874)	0.03 *
**Tumor characteristics**				
Histology	0.970 (0.896–1.050)	0.45	0.972 (0.887–1.065)	0.55
PD-L1 expression	1.249 (0.935–1.669)	0.13	1.034 (0.753–1.419)	0.84
Mutation status				
KRAS positive	0.446 (0.263–0.757)	0.003 *	0.592 (0.333–1.052)	0.07
EGFR positive	0.454 (0.220–0.934)	0.03 *	0.363 (0.154–0.855)	0.02 *
BRAF positive	0.428 (0.178–1.028)	0.06	0.497 (0.180–1.367)	0.18
ALK positive	1.880 (0.177–19.951)	0.60	4.113 (0.360–46.946)	0.26
Lung cancer stage	2.246 (0.958–5.268)	0.06	0.778 (0.283–2.136)	0.63
Localization of distant metastases				
Brain	1.320 (0.641–2.721)	0.45	1.626 (0.754–3.508)	0.22
Bone	1.428 (0.833–2.450)	0.20	1.551 (0.853–2.823)	0.15
Liver	0.833 (0.399–1.737)	0.63	0.684 (0.306–1.528)	0.35
Adrenal gland(s)	0.427 (0.241–0.756)	0.004 *	0.615 (0.327–1.156)	0.13
**Treatment**				
Current	2.785 (1.560–4.972)	<0.001 *	2.560 (1.324–4.953)	0.005 *
Line of treatment	1.004 (0.557–1.811)	0.99	1.455 (0.774–2.733)	0.24
**Site of inclusion**	0.313 (0.134–0.729)	0.007 *	0.777 (0.321–1.880)	0.58

* p-value < 0.05. [a] Missing data: BMI (n = 1), ethnicity (n = 8), albumin level (n = 10), kidney function (n = 4), smoking status (n = 12), pack years (n = 46), histology (n = 4), PD-L1 expression (n = 43), KRAS status (n = 25), EGFR status (n = 23), BRAF status (n = 13), ALK status (n = 28), lung cancer stage (n = 5), brain metastases (n = 5), bone metastases (n = 5), liver metastases (n = 5), adrenal gland(s) metastases (n = 5), baseline Cyfra 21.1 levels (n = 59), baseline CEA levels (n = 60), baseline CA-125 levels (n = 66). Abbreviations: PFS, progression-free survival; OS, overall survival; HR, hazard ratio; CI, confidence interval; BMI, body mass index; ECOG PS, Eastern Cooperative Oncology Group Performance Score; COPD, Chronic Obstructive Pulmonary Disease; NSAIDs, non-steroidal anti-inflammatory drugs; PD-L1, programmed death-ligand 1; KRAS, Kirsten rat sarcoma virus; EGFR, epidermal growth factor receptor; BRAF, B-Raf Proto-oncogene; ALK, anaplastic lymphoma kinase.

References

1. Reck, M.; Popat, S.; Reinmuth, N.; De Ruysscher, D.; Kerr, K.M.; Peters, S. Metastatic non-small-cell lung cancer (NSCLC): ESMO Clinical Practice Guidelines for diagnosis, treatment and follow-up. *Ann. Oncol.* **2013**, *25*, iii27–39. https://doi.org/10.1093/annonc/mdu199.
2. Eisenhauer, E.A.; Therasse, P.; Bogaerts, J.; Schwartz, L.H.; Sargent, D.; Ford, R.; Dancey, J.; Arbuck, S.; Gwyther, S.; Mooney, M.; et al. New response evaluation criteria in solid tumours: Revised RECIST guideline (version 1.1). *Eur. J. Cancer.* **2009**, *45*, 228–247. https://doi.org/10.1016/j.ejca.2008.10.026.
3. Yin, J.; Song, Y.; Tang, J.; Zhang, B. What is the optimal duration of immune checkpoint inhibitors in malignant tumors? *Front. Immunol.* **2022**, *13*, 983581. https://doi.org/10.3389/fimmu.2022.983581.
